# Microbiota-derived IPA alleviates intestinal mucosal inflammation through upregulating Th1/Th17 cell apoptosis in inflammatory bowel disease

**DOI:** 10.1080/19490976.2025.2467235

**Published:** 2025-02-16

**Authors:** Han Gao, Mingming Sun, Ai Li, Qiaoyan Gu, Dengfeng Kang, Zhongsheng Feng, Xiaoyu Li, Xuehong Wang, Liang Chen, Hong Yang, Yingzi Cong, Zhanju Liu

**Affiliations:** a Center for IBD Research and Department of Gastroenterology, The Shanghai Tenth People’s Hospital of Tongji University, Shanghai, China; b Department of Gastroenterology, Yanan University Affiliated Hospital, Yan’an, Shaanxi, China; c Department of Gastroenterology, The Second Xiangya Hospital, Central South University, Changsha, Hunan, China; d Department of Gastroenterology, Peking Union Medical College Hospital, Chinese Academy of Medical Sciences & Peking Union Medical College, Beijing, China; e Division of Gastroenterology and Hepatology, Department of Medicine, Feinberg School of Medicine, Northwestern University, Chicago, IL, USA; f Center for Human Immunology, Feinberg School of Medicine, Northwestern University, Chicago, IL, USA

**Keywords:** Indole-3-propionic acid, mucosal inflammation, HSP70, Th1 cells, Th17 cells

## Abstract

The gut microbiota-derived metabolite indole-3-propionic acid (IPA) plays an important role in maintaining intestinal mucosal homeostasis, while the molecular mechanisms underlying IPA regulation on mucosal CD4^+^ T cell functions in inflammatory bowel disease (IBD) remain elusive. Here we investigated the roles of IPA in modulating mucosal CD4^+^ T cells and its therapeutic potential in treatment of human IBD. Leveraging metabolomics and microbial community analyses, we observed that the levels of IPA-producing microbiota (e.g. *Peptostreptococcus*, *Clostridium*, and *Fournierella*) and IPA were decreased, while the IPA-consuming microbiota (e.g. *Parabacteroides*, *Erysipelatoclostridium*, and *Lachnoclostridium*) were increased in the feces of IBD patients than those in healthy donors. Dextran sulfate sodium (DSS)-induced acute colitis and CD45RB^high^CD4^+^ T cell transfer-induced chronic colitis models were then established in mice and treated orally with IPA to study its role in intestinal mucosal inflammation *in vivo*. We found that oral administration of IPA attenuated mucosal inflammation in both acute and chronic colitis models in mice, as characterized by increased body weight, and reduced levels of pro-inflammatory cytokines (e.g. TNF-α, IFN-γ, and IL-17A) and histological scores in the colon. We further utilized RNA sequencing, molecular docking simulations, and surface plasmon resonance analyses and identified that IPA exerts its biological effects by interacting with heat shock protein 70 (HSP70), leading to inducing Th1/Th17 cell apoptosis. Consistently, ectopic expression of HSP70 in CD4^+^ T cells conferred resistance to IPA-induced Th1/Th17 cell apoptosis. Therefore, these findings identify a previously unrecognized pathway by which IPA modulates intestinal inflammation and provide a promising avenue for the treatment of IBD.

## GRAPHICAL ABSTRACT

**Figure:**
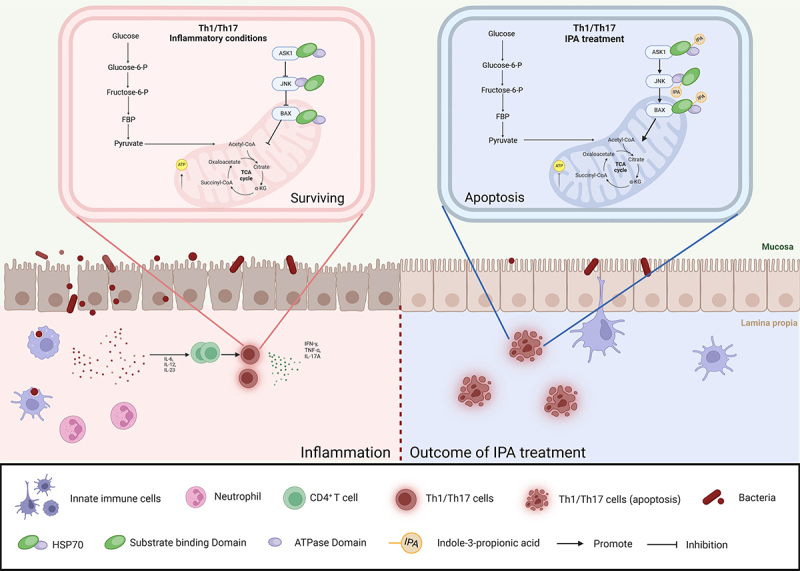
IPA promotes HSP70-mediated cell apoptosis in mucosal CD4^+^ T cells to restrain intestinal mucosal inflammation. In the context of intestinal inflammation, the content of IPA significantly diminishes in the feces of IBD patients. IPA binds to HSP70, which helps reduce mitochondrial oxidative phosphorylation of CD4^+^ T cells and induce mitochondrial apoptosis mediated by HSP70. This process causes the apoptosis of Th1/Th17 cells, thus alleviating intestinal mucosal inflammation.

## Introduction

Inflammatory bowel diseases (IBDs), including Crohn’s disease (CD) and ulcerative colitis (UC), are marked by chronic, recurring inflammation within and beyond the gastrointestinal tract.^1^ These diseases exhibit a range of complex phenotypes, primarily due to an impaired capacity to resolve incurable relapsing-remitting inflammatory damage in gut mucosa. This impaired resolution is influenced by genetic predispositions, poorly defined environmental factors, and dysbiosis of the gut microbiota.^2–4^ The intestine is a complex ecosystem that harbors a dense and diverse microbial community known as the gut microbiota, which has co-evolved with the host to establish a mutualistic relationship. Increasing lines of evidence have shown that the disruption of this fragile equilibrium, termed dysbiosis, is associated with numerous human diseases, including IBD.^5–8^ The intestinal microbiota significantly influences several key physiological functions of the host, including metabolic and nutritional homeostasis, immune system maturation and stimulation, and even brain activity.^9–11^

Multiple metabolites drive complex interactions between the host and its microbiome.^12^ The most widely studied metabolites in host-microbiota interactions are short-chain fatty acids (SCFAs), which are produced by bacterial fermentation of dietary fibers. SCFAs, including acetate, propionate, and butyrate, are essential for maintaining gut health, providing energy to colonocytes, regulating immune responses, and modulating inflammation.^13^ Bile acids, synthesized in the liver from cholesterol and secreted into the intestine, are converted from primary to secondary bile acids by the gut microbiota. This transformation impacts host metabolism, lipid digestion, and regulates signaling pathways associated with energy homeostasis and immune function.^14^ Moreover, tryptophan (Trp) metabolites, produced by the metabolism of the essential amino acid tryptophan by the host and gut microbiota, including indoles, kynurenines, and serotonin, also play critical roles in maintaining intestinal homeostasis, regulating immune responses, and affecting neurological functions.^15^ The derivative of indoles (e.g., indoleacrylic acid, indole-3-aldehyde, indoleamine 2,3-dioxygenase 1) by the gut microbiota, which is altered in patients with IBD, is crucial to intestinal homeostasis, notably through their effect on activating the aryl hydrocarbon receptor (AhR).^2^ Indole-3-propionic acid (IPA), a key metabolite produced by the intestinal microbiota, exhibits significant variances in plasma levels among patients with different diseases (e.g., anorexia nervosa and coronary artery disease).^16–18^ It functions as a reliable and efficient measure for managing unforeseen or iatrogenic ionizing radiation in clinical settings and effectively mitigating radiation-induced intestinal cancer.^19^ As a naturally occurring ligand of the AhR derived from dietary tryptophan, IPA significantly enhances polymorphonuclear myeloid-derived suppressor cell (PMN-MDSC) differentiation and suppressive effect on CD4^+^ T cells to alleviate experimental Sjögren’s syndrome.^20^ Simultaneously, IPA possesses intracellular signal transduction activity and modulates the IL-10 signaling pathway to uphold the integrity of intestinal epithelial tissue and ameliorate colitis in mice, thereby mitigating mucosal inflammation in the intestines.^21^ Nevertheless, the mechanism by which IPA regulates intestinal mucosal CD4^+^ T cell functions in IBD remains unclear and requires further investigation.

In this study, we found a significant reduction in IPA-producing microbiota and IPA but an increase in IPA-consuming microbiota in the feces of IBD patients compared to those in healthy individuals. Administration of IPA significantly attenuated both acute colitis in mice induced by dextran sulfate sodium (DSS) in drinking water and chronic colitis in mice induced by the adoptive transfer of syngeneic CD45RB^high^CD4^+^ T cells *in vivo*. Notably, IPA markedly inhibited human peripheral blood Th1 and Th17 cell differentiation *in vitro*. RNA sequencing and surface plasmon resonance techniques revealed that IPA could bind to HSP70 to facilitate Th1 and Th17 cell apoptosis, thereby alleviating intestinal mucosal inflammation, suggesting a key pathway through which IPA exerts its anti-inflammatory effects. Therefore, our data unequivocally unravel a novel mechanism whereby IPA maintains intestinal mucosal homeostasis and restrains mucosal inflammation through promoting Hsp70-mediated Th1/Th17 cell apoptosis, and provide a novel therapeutic avenue for the treatment of IBD.

## Materials and methods

### Participants and sample collections

All participants with IBD were recruited from the Center for IBD Research at the Shanghai Tenth People’s Hospital of Tongji University. Healthy controls (HC) were selected from individuals undergoing routine physical examinations at the same hospital between October 2021 and February 2024. Colon biopsies, fecal samples, and EDTA-anticoagulated blood samples were obtained from both IBD patients and HCs, including those with active Crohn’s disease (A-CD, *n* = 39), Crohn’s disease in remission (R-CD, *n* = 26), active ulcerative colitis (A-UC, *n* = 35), ulcerative colitis in remission (R-UC, *n* = 33), and HC (*n* = 59) who underwent endoscopy as part of their routine examinations (online Supplemental Table S1). Diagnoses were confirmed through clinical presentation, endoscopic and radiological examinations, and histological findings,^22–24^ following the exclusion of other diseases. Disease severity was assessed using the Crohn’s Disease Activity Index (CDAI) for Crohn’s disease and the Mayo score for ulcerative colitis, respectively.

According to the American Gastroenterological Association Institute guidelines,^25^ clinical remission was defined by a CDAI score of less than 150 points. Endoscopic severity was evaluated using the Simple Endoscopic Score for Crohn’s Disease (CD-SES) and the Ulcerative Colitis Endoscopic Index of Severity (UCEIS).^26–28^ The SES-CD included assessments of ulcers, the proportion of the surface covered by ulcers, other lesions, and stenosis. The UCEIS evaluated vascular patterns, bleeding, erosions, and ulcers. This study received approval from the Institutional Review Board for Clinical Research of the Shanghai Tenth People’s Hospital of Tongji University (SHSY-IEC-4.0/18–33/01). Written informed consent was obtained from all participants prior to the study.

### Mice

Male C57BL/6 mice and *Rag1*^−/−^ mice on a C57BL/6 background were obtained from the Shanghai Model Organisms Center (Shanghai, China) and GemPharmatech Co., Ltd. (Shanghai, China), respectively. All mice were bred and maintained under specific pathogen-free (SPF) conditions at the Experimental Animal Center of Tongji University School of Medicine (Shanghai, China). They were housed in individually ventilated cages with a 12-hour light-dark cycle and had *ad libitum* access to filtered air, sterile water, and autoclaved food. Experiments were conducted using mice aged 8 to 10 weeks. All animal experiments and procedures were approved by the Institutional Animal Care and Use Committee of Tongji University (SHDSYY-2021-1966).

### Ultra performance liquid chromatography-tandem mass spectrometry (UPLC-MS/MS)

Fecal samples were thawed on ice. Approximately 50 mg (±1 mg) of each sample was taken and homogenized with 500 μL of ice-cold methanol/water (70%, V/V) containing an internal standard. Initially, the sample was thoroughly vortexed for 3 minutes, sonicated for 10 minutes in an ice water bath, and finally vortexed for an additional 1 minute. It was then centrifuged at 12,000 rpm at 4°C for 10 minutes. After this step, 250 μL of the supernatant was transferred to a centrifuge tube, where it was centrifuged again at 12,000 rpm at 4°C for 5 minutes, and 150 μL of the supernatant was placed in the liner of the corresponding injection bottle for on-board analysis. Finally, the sample extracts were analyzed using an LC-ESI-MS/MS system (UPLC, ExionLC AD, link; MS, QTRAP® System, link; AB Sciex, Framingham, MA, USA).

### 16S rRNA sequencing and data analysis

For 16S rRNA sequencing and data analysis, fecal samples were collected from IBD patients and HC, then snap-frozen and stored at −80°C. The quality and quantity of fecal DNA were determined using NanoDrop 2000 instrument (Thermo Fisher Scientific; Waltham, MA, USA). The V4–V5 variable regions of the bacterial 16S rRNA gene were amplified via PCR with the universal primer pair 515F (5’-barcode-GTGCCAGCMGCCGCGG-3’) and 907 R (5’-CCGTCAATTCMTTTRAGTTT-3’), where the barcode represent a unique eight-base sequence for each sample. The purified PCR products were quantified using Qubit® 3.0 (Invitrogen), and every 24 amplicons with distinct barcodes were mixed in equal amounts. The pooled DNA was then utilized to create an Illumina paired-end library, following the standard procedures for genomic DNA library preparation. The resulting amplicon library was subsequently sequenced using the paired-end method (2 × 250) on an Illumina MiSeq platform (Shanghai Biozeron Co., Ltd.; Shanghai, China) in accordance with standard protocols. Finally, sequence analysis was conducted using the QIIME pipeline with default settings.

### DSS-induced acute colitis in mice

The DSS-induced acute colitis model was established in WT mice using a protocol as described previously.^29^ In brief, WT mice were provided with 2% DSS (Sigma-Aldrich; Shanghai, China) in their drinking water for 7 d, followed by a 3-day period during which the mice were given normal drinking water. The mice were monitored daily for symptoms including diarrhea, weight changes, and rectal bleeding. On day 10, all mice were sacrificed, and their spleen and colon tissues were collected for subsequent analysis.

### *T cell-transfer colitis model in* Rag1^−/−^*mice*

Splenic CD4^+^ T cells were isolated from WT mice using anti-mouse CD4 magnetic beads (BD Biosciences; San Diego, CA, USA). The isolated cells were stained with anti-mouse CD4-APC, anti-mouse CD25-PE-Cy7, and anti-mouse CD45RB-FITC monoclonal antibodies (all from BD Biosciences). Naïve CD4^+^ T cells, characterized as CD25^−^CD45RB^high^CD4^+^ T cells, were then sorted using a BD FACS Aria II Flow Cytometer (San Diego, CA, USA). A total of 5 × 10^5^ sorted cells per mouse were transferred into *Rag1*^−/−^ recipient mice via intraperitoneal injection, following the protocol as described previously.^30,31^ The recipient mice were monitored weekly for clinical signs such as weight loss, diarrhea and rectal prolapse. At the end of the 8th week, the mice were euthanized, and their large intestines were harvested for further analysis.

### Isolation of lamina propria mononuclear cells (LPMCs)

Following euthanasia, the LPMCs were isolated using an established protocol.^28^ Initially, colons were excised and sectioned into 0.5 cm segments, then thoroughly rinsed with pre-chilled phosphate-buffered saline (PBS) to remove residual fecal matter. The cleaned colon sections underwent enzymatic digestion at 37°C for 30 minutes in PBS supplemented with 5% fetal bovine serum (FBS) and 1 mm ethylenediaminetetraacetic acid (EDTA). Subsequently, the tissue was filtered, and the remaining fragments were finely minced and subjected to an additional 30-minute digestion in RPMI medium containing 5% FBS and collagenase A. The resultant supernatant, enriched with lamina propria cells, was collected and subjected to gradient centrifugation using a 40% and 75% Percoll-RPMI solution. The intermediate layer was harvested and designated as LPMCs for subsequent analyses.

### Th cell differentiation in vitro

Naïve CD4^+^ T cells were isolated from the peripheral blood of HC using anti-human naïve CD4 magnetic beads (BD Biosciences). The isolated cells (5 × 10^5^/well) were activated with plate-coated anti-human CD3 monoclonal antibody (mAb) and soluble anti-human CD28 mAb (eBioscience; Shanghai, China) in complete RPMI 1640 medium. The cells were then induced to differentiate into various T helper (Th) cell subsets: Th1 cells with IL-12 (R&D; Minneapolis, MN, USA) and anti-human IL-4 mAb (eBioscience); Th2 cells with IL-4 (R&D) and anti-human IFN-γ mAb (eBioscience); Th17 cells with a cytokine cocktail containing IL-1β (R&D), IL-6 (R&D), and IL-23 (R&D) along with anti-human IFN-γ mAb and anti-human IL-4 mAb; and inducible regulatory T cells (Tregs) with TGF-β (R&D) plus anti-human IFN-γ mAb and anti-human IL-4 mAb. These conditions ensured that naïve CD4^+^ T cells differentiated appropriately into the desired T helper cell subsets for further experimental and analytical purposes.

### RNA sequencing and data analysis

CD4^+^ T cells were isolated from both IBD patients and HC according to the above methods. CD4^+^ T cells treated with IPA were lysed with TRIzol, and total RNA was extracted according to the manufacturer’s instructions. The concentrations, purity, and integrity of the RNA were assessed, and sequencing libraries were prepared using the NEBNext® Ultra™ RNA Library Prep Kit for Illumina® (NEB; Ipswich, MA, USA), adhering to the manufacturer’s guidelines. The libraries were sequenced on an Illumina Novaseq platform, generating 150-bp paired-end reads.

### Lentivirus-mediated CD4^+^ T cell transduction

CD4^+^ T cells were isolated from human peripheral blood using anti-human naïve CD4 magnetic beads and pre-activated *in vitro* with immobilized anti-human CD3 mAb and soluble anti-human CD28 mAb for 48 hours. Subsequently, these cells (1 × 10^5^/well) were transduced with lentiviruses at a multiplicity of infection (MOI) of 180, encoding either LV-*HSPA1A*, LV-sh-*HSPA1A*, or an empty control (LV-NC), following the manufacturer’s protocols (Shanghai Genechem Co., Ltd.; Shanghai, China). After a 2-hour centrifugation, the cells were incubated with the lentiviruses for an additional 6 hours in complete RPMI 1640 medium in 24-well plates. Following two washes with RPMI 1640 medium, the transduced cells were resuspended in complete RPMI 1640 medium and further stimulated with immobilized anti-human CD3 mAb and soluble anti-human CD28 mAb for 5 days. The cells were then harvested, and total RNA was extracted to measure the mRNA and protein levels of *IFNG*, *TBET*, *TNFΑ*, *IL17A*, and *HSPA1A* using qRT-PCR and flow cytometry. Supernatants were also collected to analyze TNF-α, IFN-γ, and IL-17A production via enzyme-linked immunosorbent assay (ELISA).

### Quantitative real-time PCR (qRT-PCR)

Total RNA was extracted from cells or tissues using the TRIzol reagent (Life Technologies; Grand Island, NY, USA). Complementary DNA (cDNA) synthesis was performed with the 5 × All-in-One RT MasterMix Kit (Applied Biological Materials; Richmond, BC, Canada), following the manufacturer’s instructions. Amplification was carried out using the 7900HT Fast Real-Time PCR System (Applied Biosystems; Carlsbad, CA, USA) with the TB Green Premix Ex Taq PCR Kit (TaKaRa; Dalian, China). The qRT-PCR cycling conditions included an initial denaturation at 95°C for 30 seconds, followed by 40 cycles at 95°C for 5 seconds and 60°C for 30 seconds. All primers used for qRT-PCR analysis were detailed in online Supplemental Table S2. The relative expression levels of target genes were normalized to the housekeeping gene *GAPDH* and calculated by the 2^−ΔΔCt^ method.

### ELISA

The concentrations of serval cytokines in the culture supernatants of CD4^+^ T cells were quantified using ELISA kits (Abcam; Cambridge, UK, online Supplemental Table S3). In brief, 96-well plates were coated with capture antibodies and incubated overnight at 4°C. On the next day, an assay diluent was employed to block nonspecific binding. Subsequently, standards or sample proteins were added to the wells and incubated at room temperature for 2 h with gentle shaking. Following incubation, a detection antibody was introduced and allowed to bind for 1 h. Avidin-HRP was then added and incubated for 30 min. Finally, TMB substrate was added, and the reaction was terminated with a stop solution to develop the color. Absorbance was measured at 450 nm using a microplate spectrophotometer (BioTek; Winooski, VT, USA).

### Flow cytometry

For cell surface staining, cells were incubated with various fluorescence-labeled mAbs targeted against cellular surface markers for 30 min at 4°C. For intracellular cytokine staining, CD4^+^ T cells were isolated from the peripheral blood, spleen, or LPMCs of mice and humans. These isolated cells were stimulated with phorbol 12-myristate 13-acetate (PMA, Sigma-Aldrich) and ionomycin (Sigma-Aldrich) for 5 h in 10% FBS-RPMI medium at 37°C, with brefeldin A (eBioscience) added during the final 3 h. Cells were then fixed and permeabilized for 30 min at 4°C, followed by staining with fluorochrome-conjugated antibodies against IFN-γ, IL-17A, TNF-α, and IL-10 (all from BD Biosciences). Mitochondrial mass was assessed using Mitotracker Green and Mitotracker Deep Red, while mitochondrial membrane potential was measured using an Invitrogen kit according to the manufacturer’s instructions. After staining, cells were washed and resuspended in cold PBS for flow cytometry analysis. Detailed information on antibody concentrations and sources was provided in online Supplemental Table S3. Data acquisition was performed on a FACS Canto II (BD Biosciences) and analyzed with FlowJo software (Tree Star; Ashland, OR, USA).

### Transmission electron microscopy (TEM)

CD4^+^ T cells isolated from peripheral blood of IBD patients and healthy individuals were cultured with or without 100 μM IPA for 5 days. Following incubation, cells were fixed in an electron microscopy-grade buffer containing 2% glutaraldehyde, 2% paraformaldehyde, and 0.1 M cacodylate and stored at 4°C until electron microscopy processing. Images were obtained using a Hitachi TEM system (Tokyo, Japan). Mitochondrial quantification per cell was performed using ImageJ software, with analysis conducted by two TEM specialists blinded to the experimental conditions.

### Seahorse extracellular flux analyzer assays for glycolysis

CD4^+^ T cells isolated from peripheral blood or LPMCs were seeded into CellTak-coated Seahorse XF96 cell culture microplates (Agilent Technologies; Santa Clara, CA, USA). The extracellular acidification rate (ECAR) was measured through a Seahorse XFe96 Analyzer (Agilent Technologies). Sensor cartridges were pre-hydrated in XF calibrant solution overnight in a 37°C CO_2_-free incubator. Following pre-hydration, the cartridges were loaded with glucose (10 mm, Port A), oligomycin (1 μM, Port B), and 2-deoxyglucose (50 mm, Port C) for sequential injection. The data were analyzed using the XF Glycolysis Stress Test Report Generator (Agilent Technologies).

### Seahorse extracellular flux analyzer assays for oxidative phosphorylation (OXPHOS)

The assay was performed as described previously, with modifications to the XF assay medium, which was supplemented with 2 mm glutamine, 1 mm sodium pyruvate, and 10 mm glucose. The sensor cartridge was sequentially loaded with oligomycin (1 μM, Port A), FCCP (1 μM, Port B), and rotenone/antimycin A (0.5 μM, Port C). The data were analyzed by the XF Mito Stress Test Report Generator (Agilent Technologies).

### Western blotting

Immunoblotting was performed as described previously.^32^ CD4^+^ T cells from the LPMCs were obtained from *Rag1*^−/−^ mice. The primary antibodies used included anti-Hsp70 (Cell Signaling Technologies; Danvers, MA, USA), anti-P-Ask1 (Cell Signaling Technologies), anti-Ask1 (Cell Signaling Technologies), anti-P-Jnk (Cell Signaling Technologies), anti-Jnk (Invitrogen; Waltham, MA, USA), anti-Procaspase-9 and Cleaved caspase-9 (Abcam; Cambridge, UK), anti-procaspase-3 and cleaved caspase-3 (Cell Signaling Technologies), and anti-Actin (Santa Cruz Biotechnology; Santa Cruz, CA, USA). The membrane was then incubated with HRP-conjugated secondary antibodies and scanned using the Amersham Imager 600 ECL system (GE Healthcare; Chicago, IL, USA).

### Statistical analysis

The data were processed and analyzed by GraphPad Prism 9 software (GraphPad Software Inc.; San Diego, CA, USA). Statistical significance was determined using unpaired or paired Student’s *t*-tests and one-way analysis of variance (ANOVA). The data were presented as mean ± SEM, with *p* < 0.05 considered statistically significant. Asterisks in all figures indicate *p*-values as follows: *p < 0.05, **p < 0.01, ***p < 0.001, and ****p < 0.0001.The sample size (n) was stated in the figure legends to indicate the number of biologically independent replicates used for statistical analyses.

## Results

### Tryptophan-metabolizing microbiota and metabolites are associated with the development of IBD

**Figure 1.**
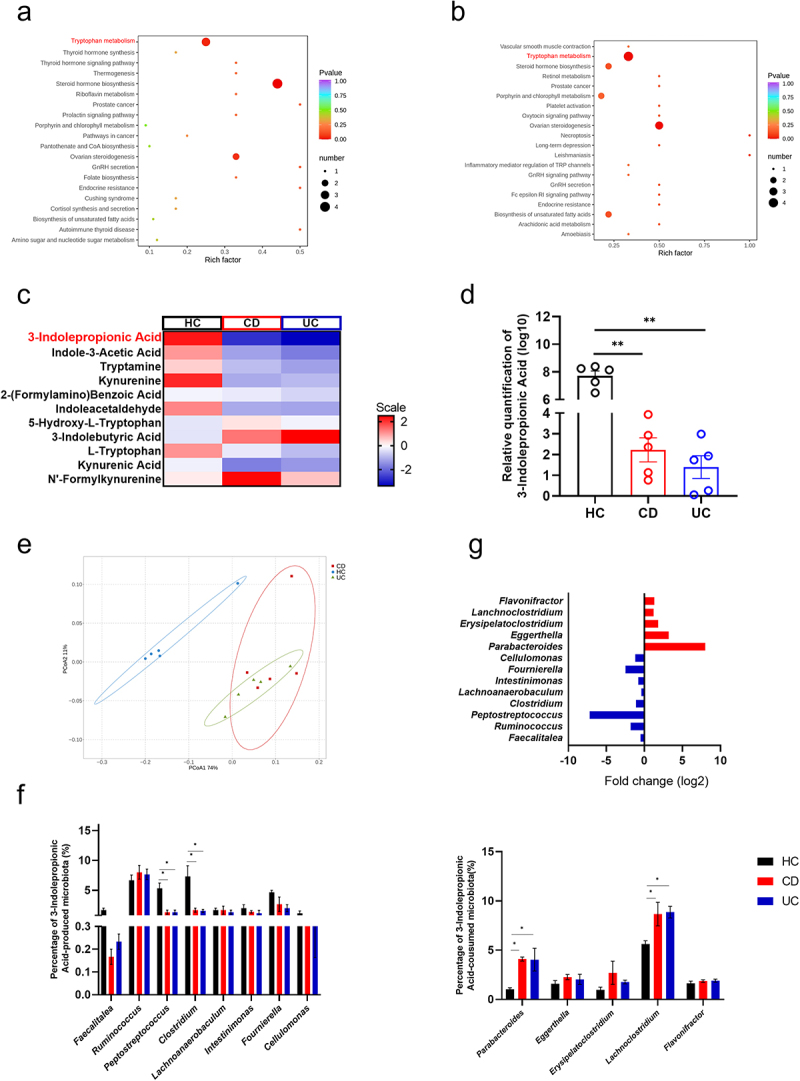
Signature alterations in tryptophan-metabolizing microbiota and metabolites in the feces of IBD patients. (a), (b) KEGG pathway enrichment analysis of differentially accumulated metabolites. (a) Pathway enrichment in CD vs. healthy individuals. (b) Pathway enrichment in UC vs. healthy individuals. (c) Comparison of tryptophan metabolite levels in the feces of IBD patients and healthy individuals. (d) Relative content of IPA in IBD patients and healthy individuals. (e) PCoA plots of the bacterial communities (16S rRNA gene amplicons) in IBD patients and healthy individuals. (f) Relative abundance of IPA-producing and IPA-consuming bacteria in the feces of IBD patients and healthy individuals. (g) Fold changes in IPA-producing microbiota (blue) and IPA-consuming microbiota (red) in the feces of IBD patients compared with healthy controls. *p < 0.05, **p < 0.01, ***p < 0.001.

To clarify the tryptophan-metabolizing microbiota and metabolites in the fecal samples of IBD patients, we collected the fecal samples from active IBD patients (CD, *n* = 5; UC, *n* = 5) and HC (*n* = 5) and conducted metabolomics and microbial community analyses using Ultra performance liquid chromatography-tandem mass spectrometry (UPLC-MS/MS) and 16S ribosomal RNA (rRNA) sequencing, respectively. KEGG pathway enrichment analysis between IBD and healthy controls revealed a significant enrichment in the metabolites of tryptophan metabolism, steroid hormone biosynthesis, ovarian steroidogenesis, biosynthesis of unsaturated fatty acids, and other pathways (Figure 1(a,b)). Notably, a marked decrease in tryptophan metabolites such as IPA, indole-3-acetic acid, tryptamine, indole acetaldehyde, 3-indolebutyric acid, and kynurenine was observed in the feces of active IBD patients (Figure 1(c,d)). Further examination of the fecal microbiota revealed a decrease in the abundance of IPA-producing microbiota (e.g., *Peptostreptococcus*, *Clostridium*, and *Fournierella*)^33–35^ but an increase in the abundance of IPA-consuming microbiota (e.g., *Parabacteroides*, *Erysipelatoclostridium*, and *Lachnoclostridium*)^36–38^ in the feces of active IBD patients (Figure 1(e-g)). These findings underscore the critical role of specific gut microbial populations in modulating IPA levels in the feces of IBD patients, thus highlighting the potential role of IPA in the pathogenesis of IBD.

### IPA ameliorates DSS-induced acute colitis in mice

**Figure 2.**
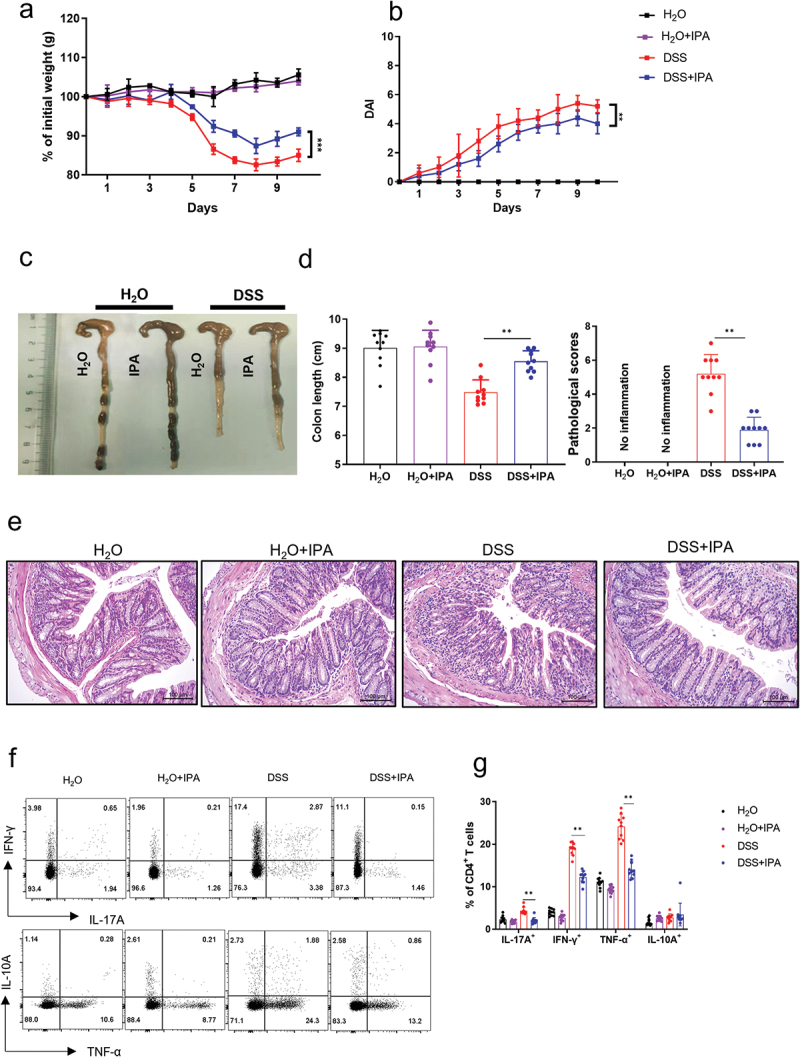
Oral administration of IPA alleviates DSS-induced acute colitis in mice. DSS-induced acute colitis was performed in WT (*n* = 10) mice. Mice were given IPA (50 mg/kg, prepared in 1 M NaOH, final pH 7.0) by oral gavage daily from 0 to 10 days of DSS treatment. An equivalent volume of H_2_O vehicle was served as control. All mice were sacrificed on day 10. (a) Changes in body weight over a 10-day experimental period were indicated as a percentage of the original weight at the start of the experiment. (b) Changes in the disease activity index (DAI) in four groups during an experimental period were recorded and shown in the chart. (c) Gross morphology of the large bowels on day 10 after DSS exposure. (d) Colon length was measured and recorded. The pathological scores of colonic sections were shown in the chart. (e) Histological appearance of colonic sections after H&E staining. Scale bars: 100 μm. (f) LPMCs were separated from WT mice receiving DSS treatment, and flow cytometric analysis was performed to determine the intracellular expression of TNF-α, IFN-γ, IL-10, and IL-17A. (g) Percentages of TNF-α^+^CD4^+^ T cells, IFN-γ^+^CD4^+^ T cells, and IL-17A^+^CD4^+^ T cells are shown in the bar chart. *p < 0.05, **p < 0.01, ***p < 0.001.

To explore the therapeutic efficacy of IPA in mitigating intestinal inflammation, we established acute colitis model in mice induced by DSS. Mice were administered 2% DSS in drinking water for 7 days, followed by a 3-day remission period with water. Throughout the 10-day protocol, mice received oral gavage of IPA daily at a dose of 50 mg/kg. Compared to controls, IPA-treated mice exhibited less weight loss, significantly lower disease activity index (DAI) scores, and reduced intestinal mucosal damage (Figure 2(a–e)). LPMCs were isolated from the colonic tissues, and no significant differences were observed in the populations of B cells and CD8^+^ T cells, while there was a notable decrease in CD4^+^ T cells in IPA-treated mice (Supplementary Figure. S1). Further analysis of LP CD4^+^ T cells using intracellular staining and flow cytometry revealed a significant reduction in the numbers of IFN-γ^+^CD4^+^, IL-17A^+^CD4^+^, and TNF-α^+^CD4^+^ T cells in IPA-treated group (Figure 2(f,g)). Additionally, the mRNA levels of *Ifng*, and *Tnfa* were found to be significantly downregulated in colon tissues (Supplementary Figure S2a). Following immunofluorescence staining of colon tissues showed markedly increased expression of *Occludin* in the IPA-treated group (Supplementary Figure S2b). Taken together, these results indicated that IPA exerts a protective effect against DSS-induced acute colitis by regulating mucosal immune response and maintaining intestinal mucosal barrier integrity.

### IPA alleviates CD4^+^ T cell transfer-induced chronic colitis in mice

**Figure 3.**
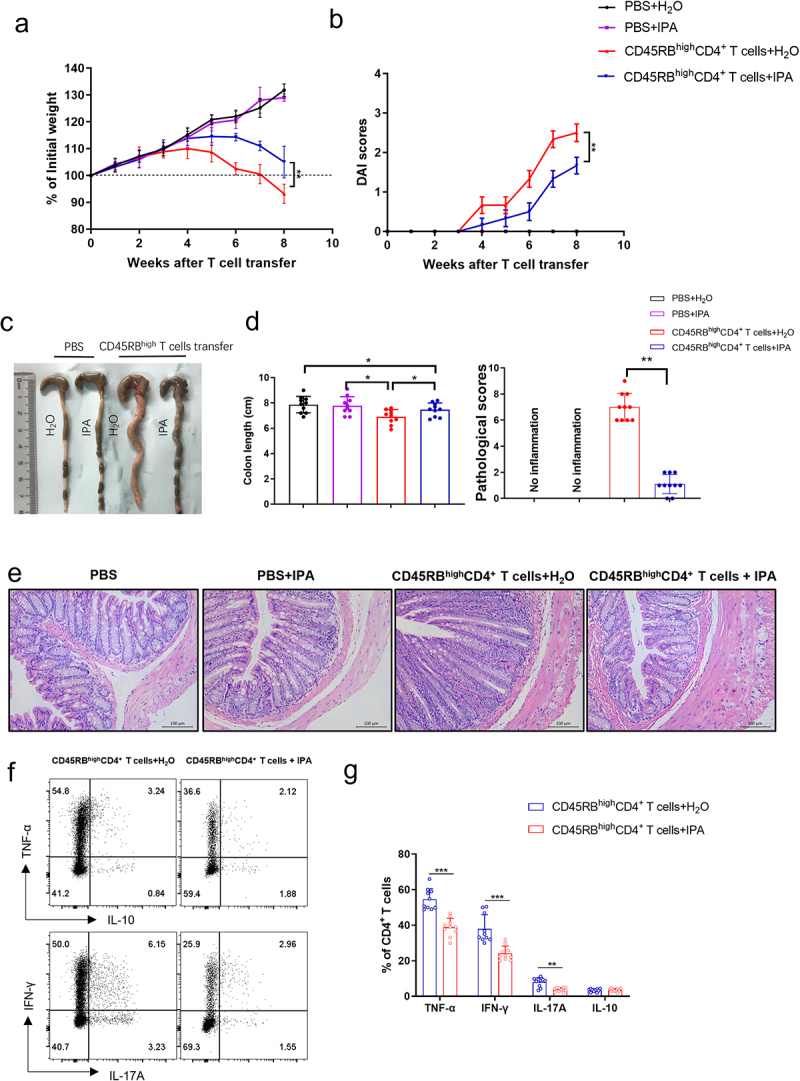
Oral administration of IPA effectively ameliorates chronic colitis in mice induced by the adoptive transfer of CD45RB^high^CD4^+^ T cells. Splenic CD45RB^high^CD4^+^ T cells were isolated from WT mice by utilizing flow cytometry and cell sorting and injected intraperitoneally into 8-week-old *Rag1*^–/–^ mice (5 × 10^5^ cells/mouse, *n* = 10/group). Mice received IPA (50 mg/kg, prepared in 1 M NaOH, final pH 7.0) via oral gavage every other day throughout the entire experimental process. An equivalent volume of H_2_O vehicle was served as control. (a) and (b) Mice were weighed weekly and DAI was calculated after T cell transfer. (c) and (d) Mice were sacrificed at week 8 after T cell transfer, and colon morphology and length were shown. (e) Pathological scores of the colonic tissues were calculated, and representative colon sections were stained with H&E. Scale bars: 100 μm. (f) LPMCs were harvested from the colons of *Rag1*^–/–^ mice transferred with CD45RB^high^CD4^+^ T cells, and intracellular expression of IL-17A, IFN-γ, and TNF-α in LP-CD4^+^ T cells was analyzed by flow cytometry. (g) Percentages and numbers of IL-17A^+^CD4^+^, IFN-γ^+^CD4^+^, and TNF-α^+^CD4^+^ T cells were shown in the chart. Statistical analyses were performed with unpaired Student’s *t*-tests. The data were presented as mean ± SEM. The data were representative of three independent experiments. Statistical analysis was performed using Tukey’s multiple comparison test (a), (b), and (d) and the unpaired Student’s *t*-test. (d) and (g). *p < 0.05, **p < 0.01, ***p < 0.001.

Confirming the potential of IPA in mitigating intestinal mucosal inflammation in mice via modulation of CD4^+^ T cells is of paramount importance. To this end, we established a chronic colitis model in *Rag1*^−/−^ mice reconstituted with syngenetic CD45RB^high^ CD4^+^ T cells and administered IPA or water orally every other day via gavage. At the eighth week, the mice were sacrificed. A marked reduction in colon inflammation was observed in IPA-treated group compared to controls, as characterized by slight decrease in the percentage of body weight and total colon length, and lower DAI score and histological scores (Figure 3(a–e)). Furthermore, IPA-treated group demonstrated a significant decline in the mRNA levels of *Tnfa*, *Tbet*, *Il17a*, *and Ifnr* in colon tissues. Conversely, no significant differences were detected in the mRNA levels of *Il4*, *Foxp3*, and *Il6* in colon tissues (Supplementary Figure S3). The frequencies of IFN-γ^+^CD4^+^, IL-17A^+^CD4^+^, and TNF-α^+^CD4^+^ T cells within LPMCs of IPA-treated mice were significantly reduced, aligning with the results by qRT-PCR (Figure 3(f,g)). Therefore, our data suggest that IPA effectively reduces colon inflammation in mice by regulating CD4^+^ T cell function.

### IPA effectively suppresses Th1/Th17 cell differentiation in IBD

**Figure 4.**
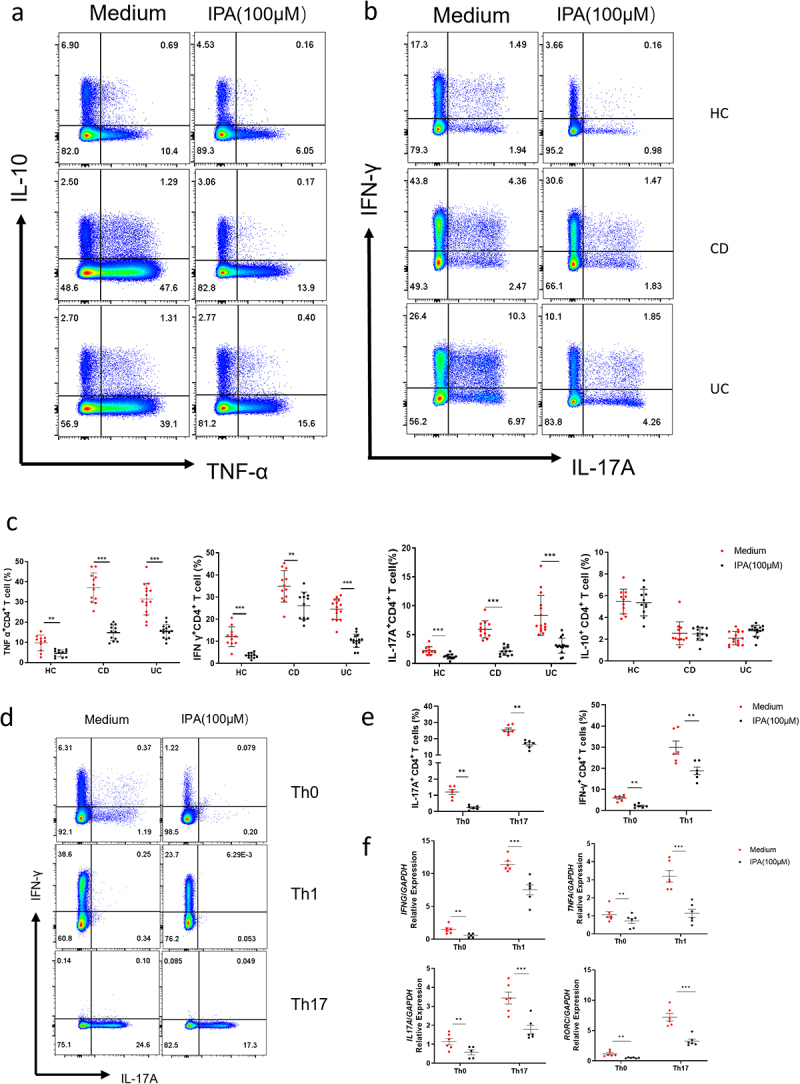
IPA compromises Th1 and Th17 cell differentiation in IBD. Peripheral blood CD4^+^ T cells isolated from healthy individuals (*n* = 11), CD patients (*n* = 12), and UC patients (*n* = 15) were stimulated *in vitro* with IPA (100 μM) for 2 or 5 days. The expression of inflammatory cytokines in CD4^+^ T cells was analyzed via qRT-PCR and flow cytometry analysis, respectively. (a-c) Expression of inflammatory cytokines in peripheral blood CD4^+^ T cells from healthy individuals, CD patients, and UC patients. (d) Peripheral blood CD4^+^ T cells from healthy individuals (*n* = 6) were isolated and cultured under different polarizing conditions. The expression of inflammatory cytokines was assessed. (e) Statistical analysis of the terminally differentiated CD4^+^ T cells in healthy individuals. (f) The expression of inflammatory cytokines in differentiated cells was measured by qRT-PCR. *p < 0.05, **p < 0.01, ***p < 0.001.

To evaluate the regulatory effect of IPA on CD4^+^ T cell functionality in IBD patients, peripheral blood samples were obtained from 53 patients with active IBD and 20 healthy subjects (detailed participant information shown in Supplementary table S1). To elucidate the regulatory role of CD4^+^ T cells throughout the study, CD4^+^ T cells were then stimulated *in vitro* using immobilized anti-CD3 and soluble anti-CD28 mAbs as indicated in the presence of IPA (100 μM) for a period of 5 days. As expected, a significant reduction in the percentages of IFN-γ-, TNF-α-, and IL-17A-positive cells was noted, whereas the percentage of IL-10-expressing cells remained unchanged (Figure 4(a–c)). Additionally, neither *FOXP3*, *IL4*, nor *GATA3* expression was influenced in CD4^+^ T cells following IPA stimulation (Supplementary Figure S4d). Consistently, the quantities of signature cytokines (e.g., IL-17A, IFN-γ, and TNF-α) were also diminished in the supernatants of CD4^+^ T cells, while production of IL-10 and IL-4 was not changed (Supplementary Figure S4e). Collectively, our data indicated that IPA may modulate immune responses by reducing Th1/Th17 cell differentiation.

To further clarify the immunomodulatory role of IPA in suppressing CD4^+^ T cell differentiation, we isolated CD4^+^ T cells from healthy donors and cultured *in vitro* for 5 days under conditions that promoted differentiation into Th0, Th1, Th2, Th17, and Treg cells, respectively. Intriguingly, a notable decrease in the frequencies of IFN-γ- and IL-17A-positive CD4^+^ T cells was observed in IPA-treated groups compared to controls under Th1- and Th17-polarization conditions, respectively (Figure 4(d,e)). However, IPA treatment had no any impact on the expression of *IL4* and *FOXP3* under Th2 and Treg cell-specific differentiation conditions, respectively (Supplementary Figure s4a and b). Additionally, qRT-PCR further revealed reduced expression levels of *IFNG*, *TNFA*, *IL17A*, and *RORC* in CD4^+^ T cells upon IPA treatment relative to the controls under identical conditions (Figure 4(f)), while *IL-10* and *IL-4* expression remained unchanged (Supplementary Figure S4d). Therefore, these data suggest that IPA may play a crucial role in diminishing Th1 and Th17 cell immune responses.

### IPA decreases HSP70 expression in CD4^+^ T cells from IBD patients

**Figure 5.**
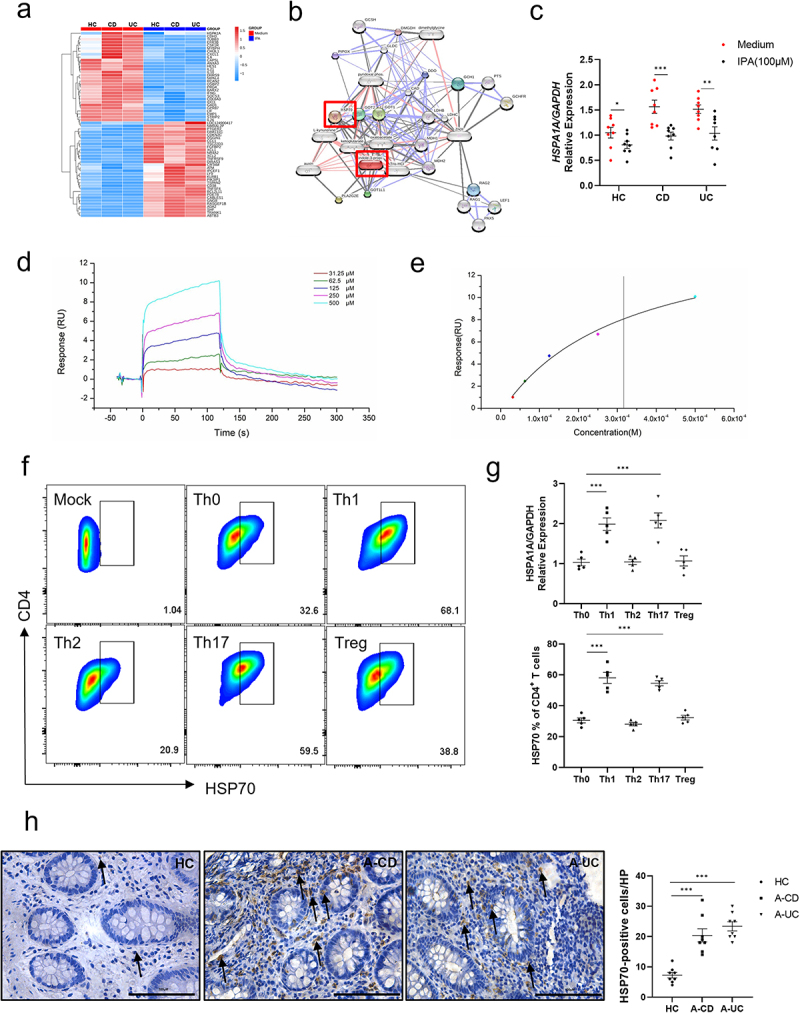
IPA decreases HSP70 expression in CD4^+^ T cells from IBD patients. CD4^+^ T cells were isolated from peripheral blood of IBD patients and healthy individuals (*n* = 3/group) and sequenced following IPA treatment. (a) The heat map illustrates the differentially expressed genes between IPA-treated and untreated groups. (b) Target genes with a Spearman correlation coefficient greater than 0.8 in RNA-seq data and the IPA potential binding protein database. (c) The expression of *HSPA1A* mRNA in peripheral blood CD4^+^ T cells from IBD patients and healthy individuals (*n* = 8/group) was detected by qRT-PCR. (d, e) Surface plasmon resonance analysis revealed that IPA binds to HSP70 protein. (f) T cells isolated from peripheral blood of five healthy individuals were cultured under Th0-, Th1-, Th2-, Th17- and Treg-polarizing conditions, respectively, for 5 days, and HSP70 expression was assessed by flow cytometry assay. (g) CD4^+^ T cells isolated from peripheral blood of five healthy individuals were cultured under Th0-, Th1-, Th2-, Th17- and Treg-polarizing conditions, respectively, for 5 days, and *HSPA1A* mRNA expression was quantified using qRT-PCR. (h) Immunohistochemistry demonstrated HSP70 staining in the colonic mucosa of healthy individuals and active IBD patients (original magnification: ✕200), and quantified the number of HSP70^+^ cells per HP field. The data were represented as the mean ± SEM. Statistical analyses were performed using Spearman correlation analysis (b), unpaired Student’s *t*-test, two-way ANOVA, Tukey’s multiple comparison test (c), and ordinary one-way ANOVA (g and h). *p < 0.05, **p < 0.01, ***p < 0.001.

**Figure 6.**
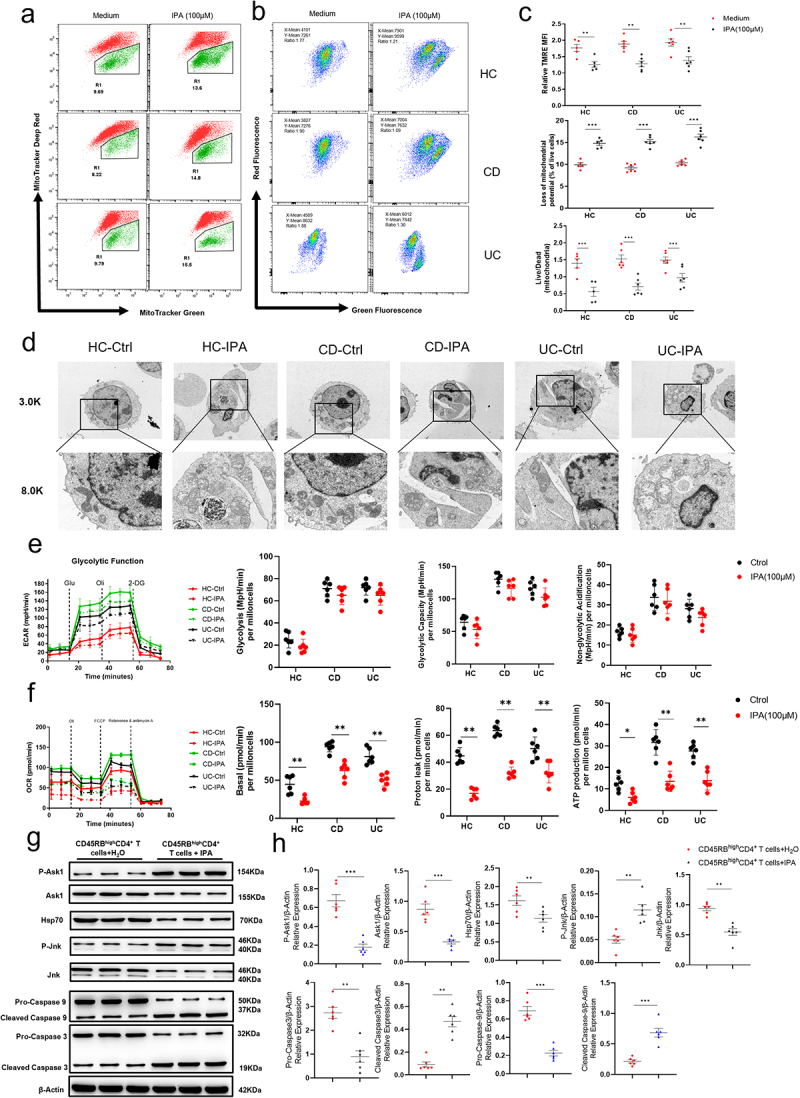
IPA disrupts mitochondrial integrity and promotes apoptosis in CD4^+^ T cells. (a-c) CD4^+^ T cells from peripheral blood of IBD patients and healthy individuals were stimulated *in vitro* with IPA (100 μM) for 5 days. Cells were collected and stained with Mito-Tracker Green, Mito-Tracker Deep Red (a), or JC-1 (b) for 30 minutes. The stained cells were then analyzed by flow cytometry, and the results were statistically summarized in (c). (d) Representative scanning electron microscopy images of CD4^+^ T cells and their mitochondria after IPA stimulation. The images were taken at magnifications of × 3.0k (upper panel) and × 8.0k (lower panel). (c) Based on the data from (d), a quantitative analysis was performed to determine the ratio of viable mitochondria to apoptotic mitochondria per field of view. (e) The dynamic changes in glycolysis of IPA-treated CD4^+^ T cells from peripheral blood of IBD patients and healthy individuals were measured using a Seahorse extracellular flux analyzer. (f) The dynamic changes in mitochondrial stress of IPA-treated CD4^+^ T cells from peripheral blood of IBD patients and healthy individuals were also assessed using a Seahorse extracellular flux analyzer. (g) Western blot analysis of CD45RB^high^CD4^+^ T cells treated with or without IPA *in vivo* showed the expression levels of P-Ask1, Ask1, Hsp70, P-Jnk, Jnk, Pro-Caspase 9, Cleaved Caspase 9, Pro-Caspase 3, and Cleaved Caspase 3 caspase, respectively. Statistical analyses, including unpaired Student’s *t*-test, two-way ANOVA, and ordinary one-way ANOVA, were performed to validate these observations. The data were presented as the mean ± SEM, with significance indicated by *p < 0.05, **p < 0.01, and ***p < 0.001.

To clarify the essential role of IPA in regulating CD4^+^ T cell functionality, these cells were isolated from human peripheral blood and subjected to *in vitro* stimulation with IPA, followed by RNA sequencing analysis. As shown in Figure 5(a), the expression of proinflammatory genes (e.g., *ANXA3*, *IL21*, and *GPR87*) and the tryptophan metabolism-related gene *IDO1* markedly decreased in IPA-treated group. Although previous studies have demonstrated that IPA activates the AhR and pregnane X receptor (PXR) pathways to regulate cellular immune responses,^39,40^ our sequencing results demonstrated that the mRNA levels of transcription factors associated with these two pathways were relatively low and failed to have a significant difference. Therefore, we hypothesize that IPA may regulate T cell immune function through an alternative pathway (Supplementary Figure S5). Further screening of the chemical structure of IPA enabled us to predict potential protein targets, and HSP70 was finally selected as a potential target as highlighted in Figure 5(b). Among the differentially expressed genes, *HSPA1A*, which encodes HSP70, merited particular attention. HSP70 is critical for protein folding, assembly, and degradation, thus ensuring intracellular protein homeostasis.^41^ Previous studies have demonstrated that HSP70 plays a pivotal role in the differentiation of Th17 cells in the progression of some autoimmune diseases (e.g., psoriasis and multiple sclerosis).^42,43^ Notably, *HSPA1A* was observed to be highly expressed in CD4^+^ T cells of IBD patients but markedly reduced following IPA stimulation in both IBD patients and healthy donors (Figure 5(c)). To elucidate the molecular mechanisms underlying the regulation of IPA on HSP70, we conducted a series of assays. Surface plasmon resonance analysis revealed that IPA was directly bound to HSP70 (Figure 5(d,e)). Data from the Human Protein Atlas revealed that HSP70 expression is notably high in epithelial cells but relatively low in neutrophils, T cells and macrophages.^44^ We then analyzed HSP70 protein levels in various differentiated T cell subsets (i.e., Th1, Th2, Th17, and Treg cells) and found that Th1 and Th17 cells exhibited significantly higher levels of HSP70 expression compared with Th0, Th2, and Treg cells, respectively (Figure 5(f,g)). Immunohistochemical analysis of intestinal mucosal sections further revealed a significant upregulation of HSP70 in inflamed mucosa of IBD patients relative to healthy controls (Figure 5(h)). Moreover, oral administration of IPA also resulted in a marked decrease in HSP70 protein expression in LP CD4^+^ T cells from a chronic colitis mouse model induced by the adoptive transfer of CD45RB^high^CD4^+^ T cells (Figure 6(g)).

### IPA promotes Th1 and Th17 cell apoptosis through restraining HSP70

Considering the role of HSP70 in regulating mitochondrion-dependent apoptotic pathways, we hypothesized that IPA might mitigate colonic inflammation progression by inducing CD4^+^ T cell apoptosis through downregulation of HSP70 activity. Accordingly, we assessed mitochondrial activity and mitochondrial membrane potential in IPA-stimulated CD4^+^ T cells, showing that IPA stimulation did promote apoptosis (Figure 6(a–c)). We also assessed mitochondrial activity in LP-CD4^+^ T cells isolated from chronic colitis mouse model induced by the adoptive transfer of CD45RB^high^CD4^+^ T cells following oral IPA treatment, showing a significant reduction in mitochondrial activity in IPA-treated CD4^+^ T cells (Supplementary Figure S6a and b). Transmission electron microscopy examination of these cells post-IPA stimulation uncovered increased mitochondrial swelling and compromised mitochondrial membrane architecture (Figure 6(d)). Simultaneously, we tested the glycolytic capacity and mitochondrial oxidative phosphorylation of human CD4^+^ T cells and found that IPA stimulation had no impact on the glycolytic capacity of these cells but significantly impaired their mitochondrial oxidative phosphorylation (Figure 6(e,f)). In accordance with our RNA sequencing data, the mRNA levels of genes associated with the glycolysis pathway also exhibited no significant differences before and after IPA treatment (Supplementary Figure S5). We then isolated LP CD4^+^ T cells from inflamed colon of CD45RB^high^CD4^+^ T cell-induced chronic colitis model and conducted glycolytic and mitochondrial stress tests, showing that the glycolytic function of CD4^+^ T cells remained unchanged in IPA-treated group, whereas the level of oxidative phosphorylation was reduced (Supplementary Figure S6c and d). Interestingly, HSP70 inhibited cell apoptosis by suppressing the phosphorylation of ASK1 and JNK. Consistently, a significant increase in the phosphorylation levels of ASK1 and JNK was seen in IPA-treated CD4^+^ T cells (Figure 6(g)). Taken together, these data imply that IPA does induce CD4^+^ T cell apoptosis by attenuating HSP70 activity.

**Figure 7.**
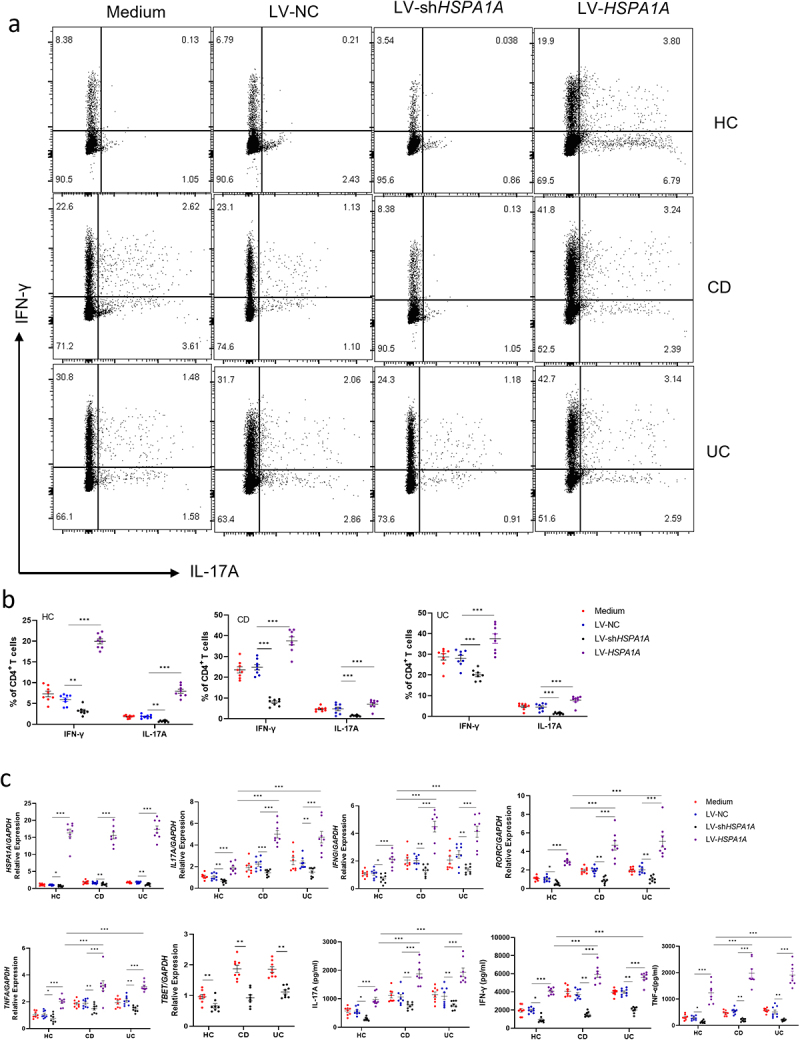
Overexpression of *HSPA1A* promotes Th1/Th17 cell immune response in IBD patients and healthy individuals. (a, b) Peripheral blood CD4^+^ T cells (1 × 10^5^ cells/well) were isolated from 8 healthy individuals, 8 active Crohn’s disease patients, and 8 active ulcerative colitis patients. The cells were then transduced with lentiviruses expressing shRNA targeting *HSPA1A* (LV-sh*HSPA1A*), *HSPA1A* (LV-*HSPA1A*), or a negative control (LV-NC). These cells were co-cultured with plate-bound anti-CD3 mAb (5 μg/mL) and soluble anti-CD28 mAb (2 μg/mL) for 5 days. The frequencies of IL-17A- and IFN-γ-expressing CD4^+^ T cells were analyzed by flow cytometry. (c) The RNA expression levels of various genes in the transduced CD4^+^ T cells were measured using qRT-PCR. The levels of inflammatory cytokines secreted by the transduced CD4^+^ T cells were detected using ELISA. The data were presented as the mean ± SEM. Statistical analyses were evaluated using Tukey’s multiple comparisons test. *p < 0.05, **p < 0.01, ***p < 0.001.

To further elucidate the role of *HSPA1A* in the regulation of CD4^+^ T cell apoptosis, we isolated PB-CD4^+^ T cells from active IBD patients and healthy controls. Cells were transfected with LV-NC, LV-sh-*HSPA1A*, or LV-*HSPA1A* according to the instructions. Cells were then collected on 2, 3, and 5 days, respectively, and the expression of various cytokines was determined using qRT-PCR, ELISA, and flow cytometry, respectively. Our data revealed that the LV-sh-*HSPA1A* transfectants significantly reduced the expression levels of *HSPA1*, *TNFA*, *IFNG*, and *IL17A* compared to controls, whereas the LV-*HSPA1A* transfectants showed significantly higher levels of these cytokines. Similarly, the same trends of TNF-α, IFN-γ, and IL-17A expression were also observed in both CD4^+^ T cells and the culture supernatants via flow cytometry and ELISA, respectively, in LV-sh-*HSPA1A*- and LV-*HSPA1A*-transfected groups (Figure 7(a–c)). To decipher the effect of *HSPA1A* on mitochondrion-dependent apoptosis, we tested mitochondrial activity in the transfected cells and observed a significant decrease in mitochondrial activity in LV-sh-*HSPA1A*-transfected group but a significant increase in LV-*HSPA1A*-transfected group compared to controls (Supplementary Figure S7a and b). Altogether, these data indicate that IPA alleviates intestinal mucosal inflammation by promoting the apoptosis of Th1 and Th17 cells through inhibiting HSP70 expression.

## Discussion

In this study, we observed that levels of gut microbiota-derived metabolite IPA were significantly lower in in the feces of IBD patients compared to healthy controls. Further analysis revealed that IPA profoundly inhibits Th1/Th17 cell-mediated immune responses. Importantly, we discovered that IPA promotes apoptosis of mucosal Th1/Th17 cells and alleviates intestinal mucosal inflammation by binding to HSP70. This was further corroborated by *in vitro* experiments, showing that down-regulated HSP70 could significantly mitigate Th1/Th17 cell-mediated immune responses. These findings highlight the critical role of IPA in reducing mucosal inflammation and may serve as a new natural therapeutic agent for human IBD.

Previous studies have shown that the gut microbiota plays a crucial role in human physiology, with many of its effects mediated by metabolites produced by the microorganisms themselves or by metabolites transformed from environmental or host molecules. Among these metabolites, those derived from the essential aromatic amino acid tryptophan have been demonstrated to regulate mucosal immunity and maintain gut homeostasis.^15,45,46^ Our findings are aligned with these observation, demonstrating that IPA was significantly lower in the feces of IBD patients than that in healthy individuals. As an intestinal microbiota metabolite, IPA can also be absorbed through the intestinal tract and enter the bloodstream. Previous study has demonstrated that the tryptophan metabolites in the serum of IBD patients are negatively correlated with the disease activity of the patients.^39^ Tryptophan metabolism involves three pathways, including host metabolic pathways (the kynurenine and serotonin pathways) and microbial metabolic pathway (indole metabolism). Specifically, *Clostridium sporogenes*, *Bacteroides species*, and *Lactobacillus reuteri* convert tryptophan into tryptamine and indolepyruvate, which are ultimately transformed into indole-3-acetic acid, indole-3-propionic acid, and indolelactic acid.^47–49^ It has been proven that the concentration of IPA in the feces of IBD patients is reduced, suggesting that the lack of tryptophan metabolites may exacerbate the symptoms of IBD.^50^ In agreement with previous reports, our results also confirmed a significant decrease in IPA in the feces of IBD patients. Importantly, IPA-producing microbiota is decreased while IPA-consuming microbiota is increased in the feces of IBD patients. Further study is warranted to elucidate the mechanisms by which IPA and other tryptophan metabolites influence gut health and explore potential interventions that could mitigate the symptoms of IBD by restoring the balance of these critical compounds.

IPA is a bacterial metabolite synthesized from tryptophan via deamination reactions by commensal gut bacteria, such as *Clostridium sporogenes*. Upon dissolution in water, IPA generates various AhR agonists subsequently and is recognized for its ability to decrease gut permeability while enhancing gut barrier function via the activation of PXR and Toll-like receptor 4 (TLR4).^51,52^ Previous studies have demonstrated that in mice with undetectable IPA levels, immune cells (e.g., neutrophils, monocytes, and memory CD4^+^ T cells) appear to be significantly increased, leading to increased intestinal epithelial cell permeability and a higher susceptibility to diseases such as IBD.^53^ Our study corroborates these findings, demonstrating that serum IPA levels was reduced in DSS-induced colitis and that oral administration of IPA could markedly alleviate gut inflammation. Previous data have proven that IPA activates AhR and significantly induces the production of IL-10R1 in cultured intestinal epithelial cells, thus promoting gut microenvironment stability.^21^ Also, the AhR pathway influences T cell differentiation and the activity of antigen-presenting cells. Recent work has shown that the AhR agonist indole-3-carbinol (ITE) induces tolerogenic properties in both human and mouse dendritic cells (DCs). ITE increases the levels of IL-10 and TGF-β but reduces the levels of IL-12 and IL-6. This shift in cytokine profiles promotes the generation of antigen-specific Foxp3^+^ regulatory T cells (Tregs) and type 1 regulatory T (Tr1) cells, thereby combating inflammation in experimental autoimmune encephalomyelitis (EAE) mouse models.^54^

Unlike observations in the EAE model, our study did not detect any changes in the number of Foxp3^+^ Tregs. Instead, we found that oral administration of IPA significantly reduced the production of Th1 and Th17 cells in LPMCs of colitis mice, thereby alleviating colonic inflammation. Intriguingly, IPA also inhibited the function of Th1/Th17 cells in human peripheral blood. These findings implied that IPA modulates immune responses through mechanisms distinct from those in the EAE model, specifically by reducing both the production and function of pro-inflammatory Th1 and Th17 cells. To further elucidate the role of IPA in modulating CD4^+^ T cells, we conducted RNA sequencing and molecular docking experiments on IPA-stimulated CD4^+^ T cells, identifying a novel binding protein for IPA, namely HSP70. HSP70, a highly conserved molecular chaperone, comprises an N-terminal ATPase domain and a C-terminal substrate-binding domain that recognizes polypeptide substrates. These two domains functionally couple with the ATPase activity of HSP70 hydrolyzing ATP to ADP, leading to conformational changes in adjacent domains, thereby enhancing substrate binding affinity.^55^ Contrary to previous findings showing a decreased expression of HSP70 in the intestinal mucosa of IBD patients under inflammatory conditions, our study revealed an increased expression of HSP70 in CD4^+^ T cells under verification. We propose that the increase in HSP70 expression in inflammatory CD4^+^ T cells may be obscured by the diminished expression in epithelial cells, especially given the substantial reduction in the number of intestinal epithelial cells during colitis.^56^ A large body of literature has suggested that HSP70 possesses diverse functions involved in the inhibition of apoptotic pathways.^17,57,58^ Therefore, our study has highlighted the critical role of orally administered IPA that reduces the proportions of Th1 and Th17 cells in LPMCs of chronic colitis mice, accompanied by activation of the HSP70-associated apoptotic pathways and a significant increase in mitochondrion-dependent apoptosis. Upon IPA stimulation of human peripheral blood CD4^+^ T cells, their glycolytic capacity remains unaffected, but mitochondrial oxidative phosphorylation capacity is significantly reduced. Additionally, ectopic expression of *HSPA1A* in peripheral blood CD4^+^ T cells promotes Th1 and Th17 cell immune responses, while silencing expression of *HSPA1A* in CD4^+^ T cells could inhibit Th1 and Th17 cell immune responses.

In summary, our data reveal that IPA is instrumental in alleviating colonic inflammation by directly binding to HSP70. This interaction affects mitochondrial function and promotes caspase-dependent apoptosis in CD4^+^ T cells, leading to the induction of mucosal Th1 and Th17 cell apoptosis and subsequent reduction in intestinal mucosal inflammation. Our study has highlighted the advancement of the therapeutic potential of IPA and provided new insight into the potential of targeting HSP70 in CD4^+^ T cells as a novel strategy for IBD treatment.

## Supplementary Material

Supplemental Material

## Data Availability

All data supporting the findings of this study are included in the article and/or the supplementary materials. The original data sets are also available from the corresponding author upon request.

## Abbreviations

IPA: Indole-3-propionic acid
IBD: Inflammatory bowel disease
DSS: Dextran sulfate sodium
HSP70: Heat shock protein 70
CD: Crohn’s disease
UC: Ulcerative colitis
CDAI: Crohn’s disease activity index
UCEIS: Ulcerative colitis endoscopic index of severity
LPMC: Lamina propria mononuclear cell
UPLC-MS/MS: Ultra performance liquid chromatography-tandem mass spectrometry
