# Supplementary material for: Microbiota-derived IPA alleviates intestinal mucosal inflammation through upregulating Th1/Th17 cell apoptosis in inflammatory bowel disease
Source: Gut Microbes. 2025 Feb 16;17(1):2467235. doi: 10.1080/19490976.2025.2467235 (PMC11834480; doi:10.1080/19490976.2025.2467235)
Supplement: Supplemental Material [file KGMI_A_2467235_SM3496.zip › Supplementary figures and method.docx]

Supplementary Materials for

**Microbiota-derived IPA alleviates intestinal mucosal inflammation through upregulating Th1/Th17 cell apoptosis in inflammatory bowel disease**

Han Gao *et al*.

*Corresponding author: Prof. Dr. Liang Chen, E-mail: [cl666a@163.com](mailto:cl666a@163.com). Prof. Dr. Hong Yang, E-mail: [hongy72@163.com](mailto:hongy72@163.com). Prof. Dr. Zhanju Liu, E-mail: Liuzhanju88@126.com

**This file includes:**

Figs. S1 to S8

Tables S1 to S3


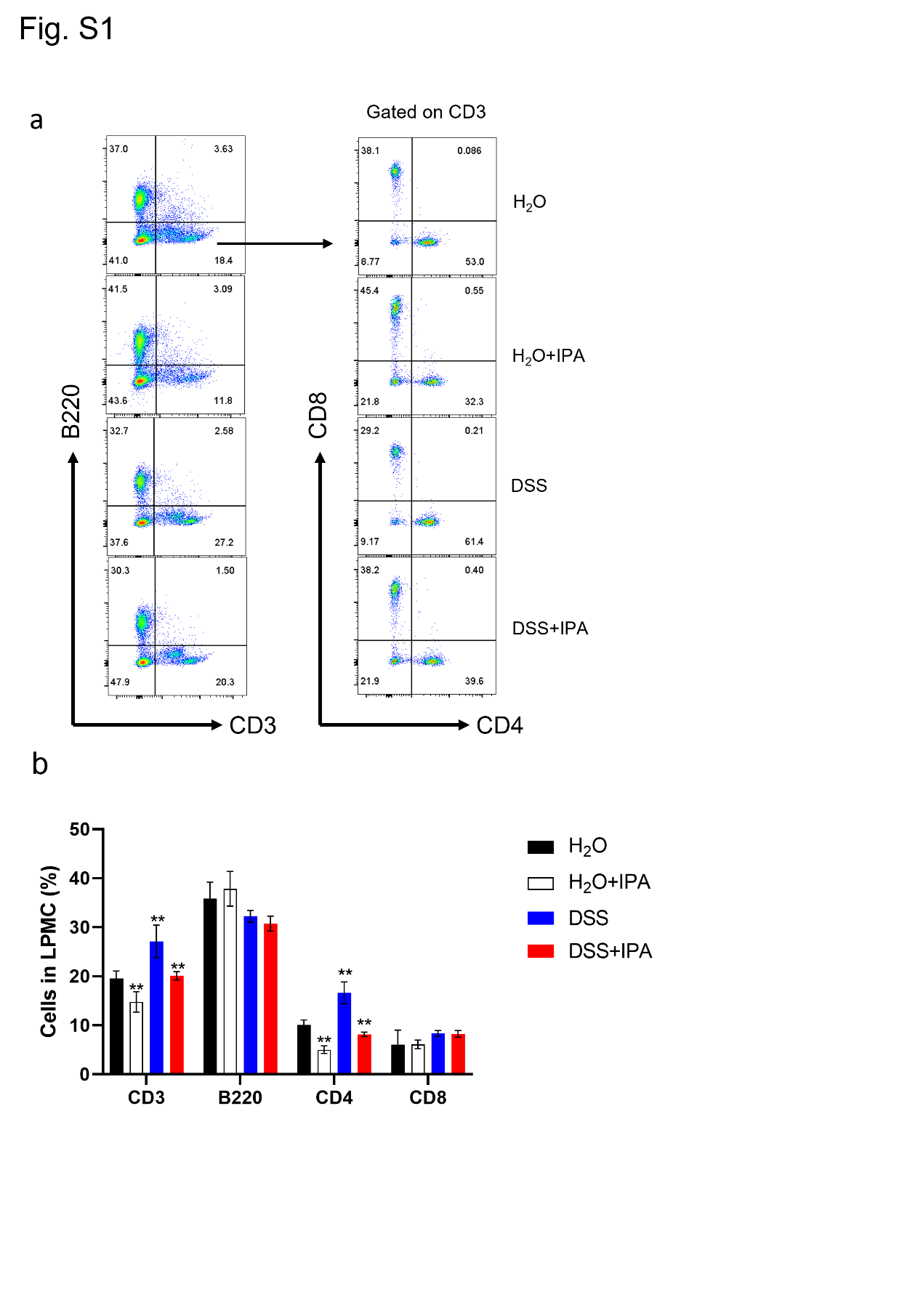


**Figure S1** Effect of IPA on CD4^+^ T cell differentiation in LPMCs *in vivo*. DSS-induced acute colitis was performed in WT mice (n = 10). Mice were given IPA (50 mg/kg, prepared in 1M NaOH, final pH 7.0) by oral gavage daily from 0 to 10 days of DSS treatment. An equivalent volume of H_2_O vehicle was served as control. (a) Single-cell suspensions of LPMCs were prepared from IPA-treated WT mice as indicated in figure 2. Flow cytometry was performed to determine cell phenotypes, including B220^+^ B cells, CD3^+^ T cells (gated on total live cells), and further subsets of CD4^+^ and CD8^+^ T cells (gated on CD3^+^ T cells). (b) The percentages of these cells within LPMCs were depicted in the bar chart. Values were expressed as mean ± SEM. Statistical significance was assessed using Student’s unpaired *t*-tests. **p* <0.05, ** *p* <0.01.


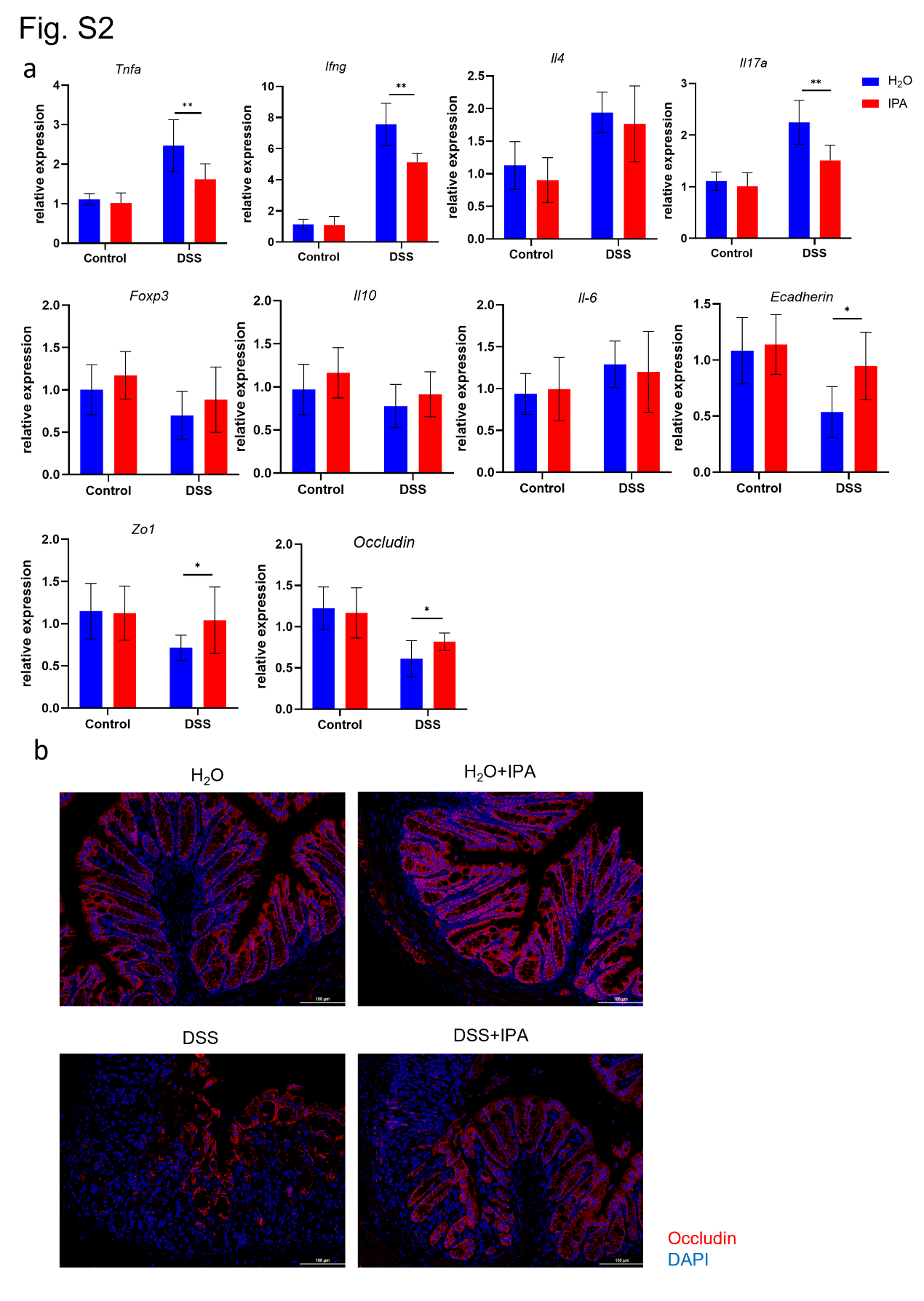


**Figure S2** Oral administration of IPA ameliorates DSS-induced acute colitis in mice. WT mice (n = 10) were subjected to DSS-induced acute colitis as indicated in Figure 2. Mice were orally administered IPA (50 mg/kg, prepared in 1M NaOH, final pH 7.0) or an equivalent volume of H_2_O as controls from 0 to 10 days of DSS treatment. All mice were sacrificed on day 10, and colon tissues were harvested for analysis. (a) The mRNA levels of pro-inflammatory cytokines *(Tnfa*, *Ifng*, *Il17a*, and *Il6)*, anti-inflammatory markers *(Il4*, *Foxp3*, and *Il10)*, and epithelial integrity markers *(E-cadherin,* *Occludin,* and *Zo1)* were quantified using qRT-PCR. (b) Representative immunofluorescent images of colonic mucosa for staining with DAPI (blue) and anti-Occludin (red). Scale bars: 100 μm. Data were expressed as mean ± SEM. Statistical analysis was performed using the student’s unpaired *t*-test. Significance is indicated as * *p* <0.05 and ** *p* <0.01 compared to controls.


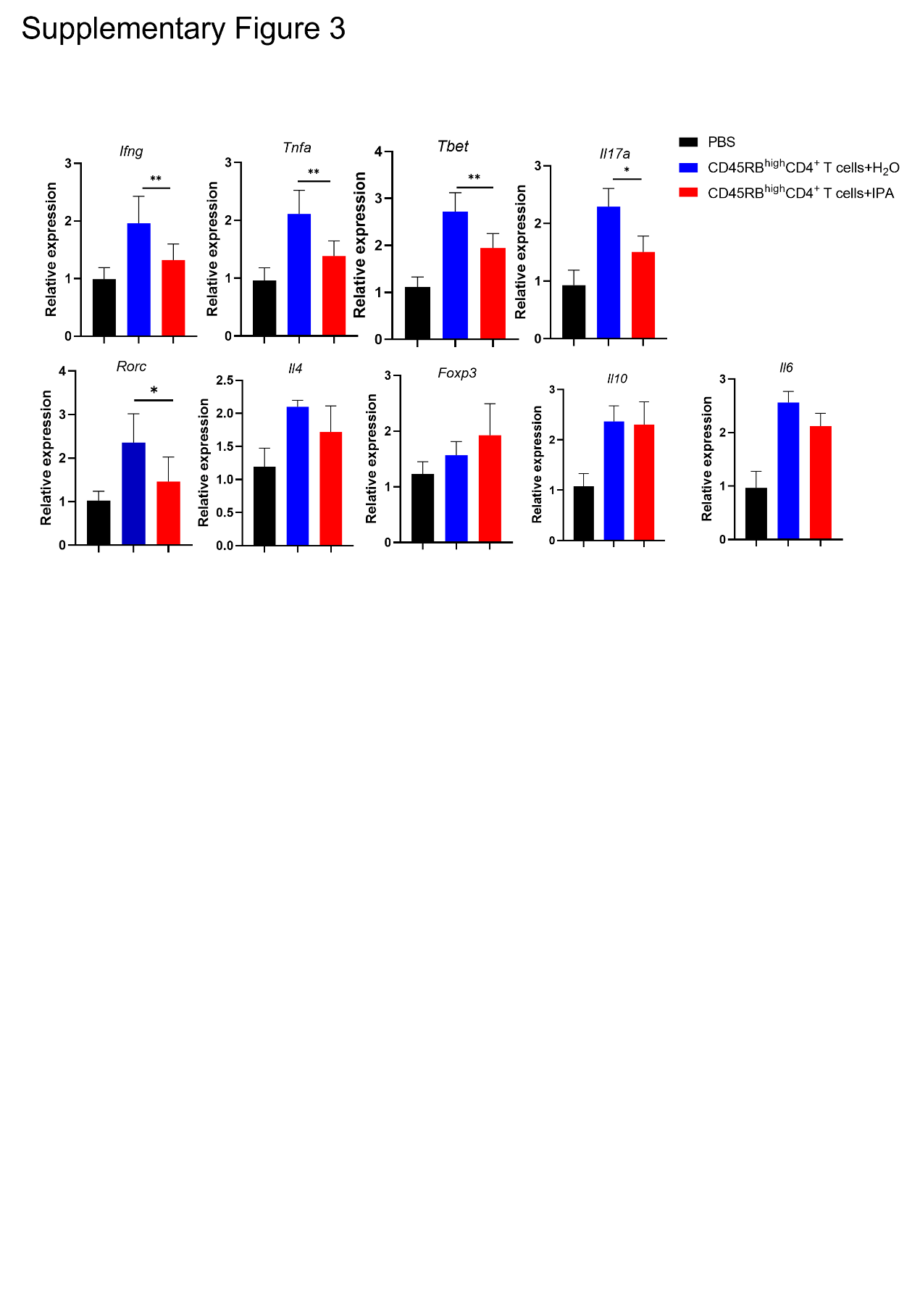


**Figure S3** Oral administration of IPA effectively reduces chronic colitis in mice. Chronic colitis model in *Rag1^-/-^* mice (n=10) were induced by adoptive transfer of CD45RB^high^CD4^+^ T cells (5×10^5^ cells/mouse) isolated from spleen of WT mice intraperitoneally, as indicated in Figure 3. Following cell transfer, mice were orally administered IPA (50 mg/kg, prepared in 1M NaOH, final pH 7.0) or an equivalent volume of H_2_O as controls via oral gavage every other day throughout the entire experimental process. The colon tissues were harvested 8 weeks after T cell transfer, and the mRNA levels of different cytokines (*Ifnr*, *Tnfa*, *Il17a*, *Il4*, *Il10*, and *Il6*) and transcription factors (*Tbet*, *Rorc*, and *Foxp3*) were assessed using qRT-PCR. Data were presented as mean ± SEM. Statistical significance was determined using Student’s unpaired *t*-test, with * *p* <0.05 and ** *p* <0.01 indicating significant differences compared to control-treated mice.


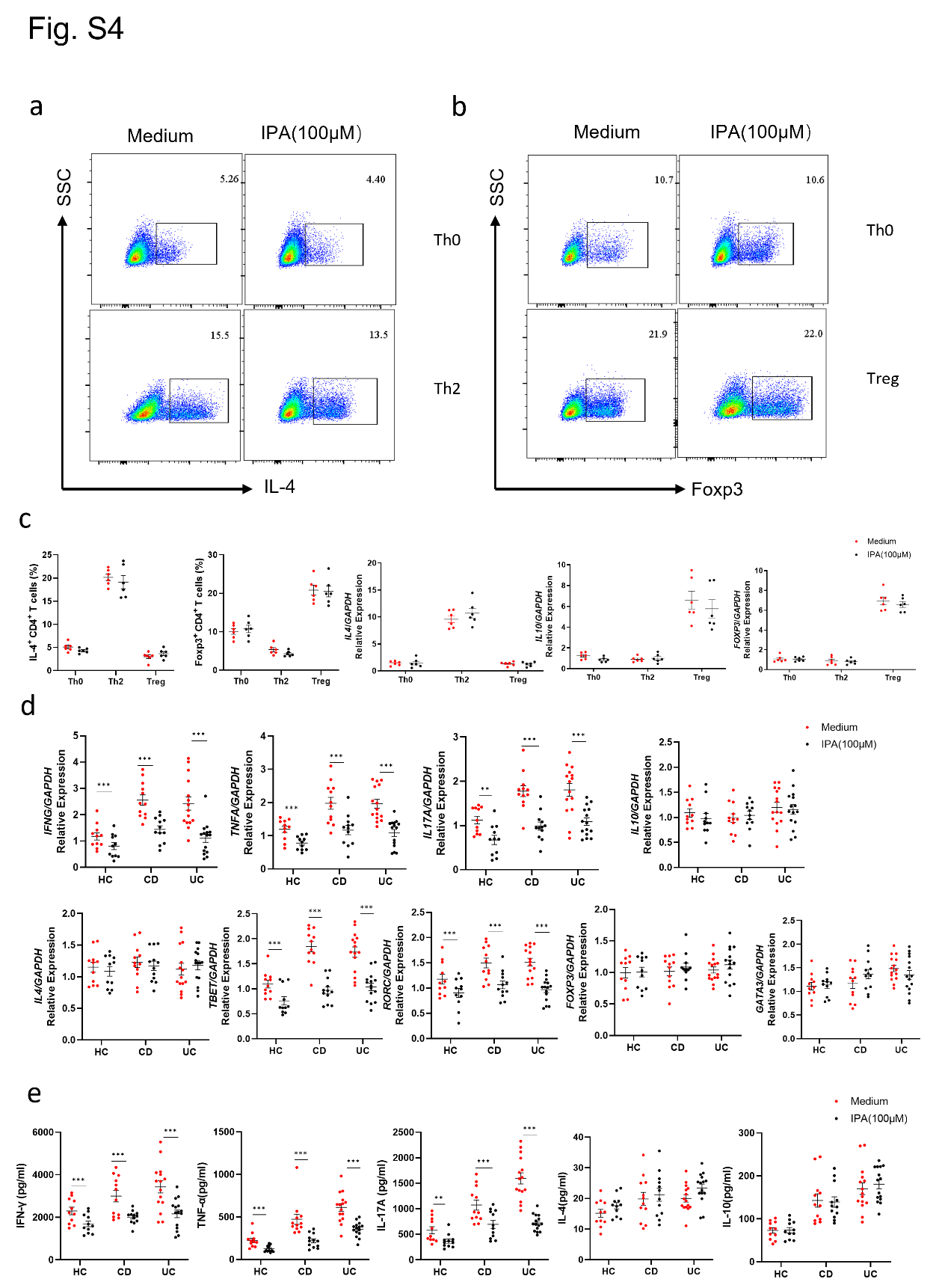


**Figure S4** IPA profoundly suppresses Th1/Th17 cell differentiation in IBD patients. Peripheral blood CD4^+^ T cells isolated from healthy individuals (n = 11), CD patients (n = 12), and UC patients (n = 15) were stimulated with IPA (100 μM) *in vitro* for 2, 3 or 5 days, and inflammatory cytokines in CD4^+^ T cells were analyzed via flow cytometry analysis, qRT-PCR, and ELISA, respectively. (a-c) Peripheral blood CD4^+^ T cells isolated from HC (n=6) were cultured under different polarizing conditions as indicated in Figure 4, and the expression of inflammatory cytokines was assessed by qRT-PCR (d) The expression of inflammatory cytokines in peripheral blood CD4^+^ T cells from all donors was measured by qRT-PCR. (e) The expression of inflammatory cytokines in peripheral blood CD4^+^ T cells from all donors was measured by ELISA. The data were presented as mean ± SEM. Statistical significance was determined using the Student’s *t*-test, with * *p* <0.05, ** *p* <0.01, and *** *p* < 0.001 indicating significant differences compared to the control.


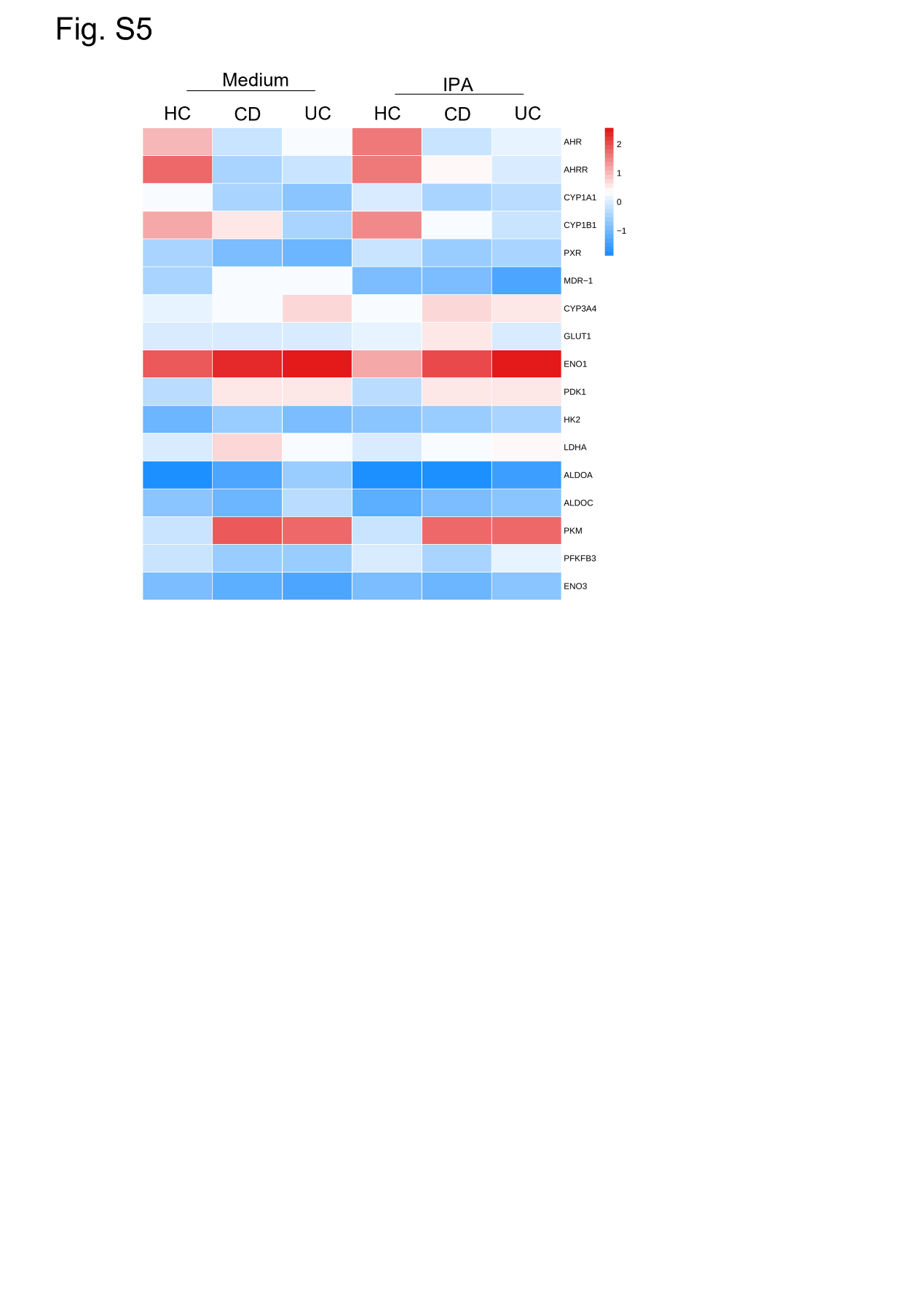


**Figure S5** CD4^+^ T cells were isolated from peripheral blood of IBD patients and healthy individuals (n = 3/group) and sequenced following IPA treatment. The heat map illustrates the expressed genes of the glycolysis pathway, AhR and PXR pathways.


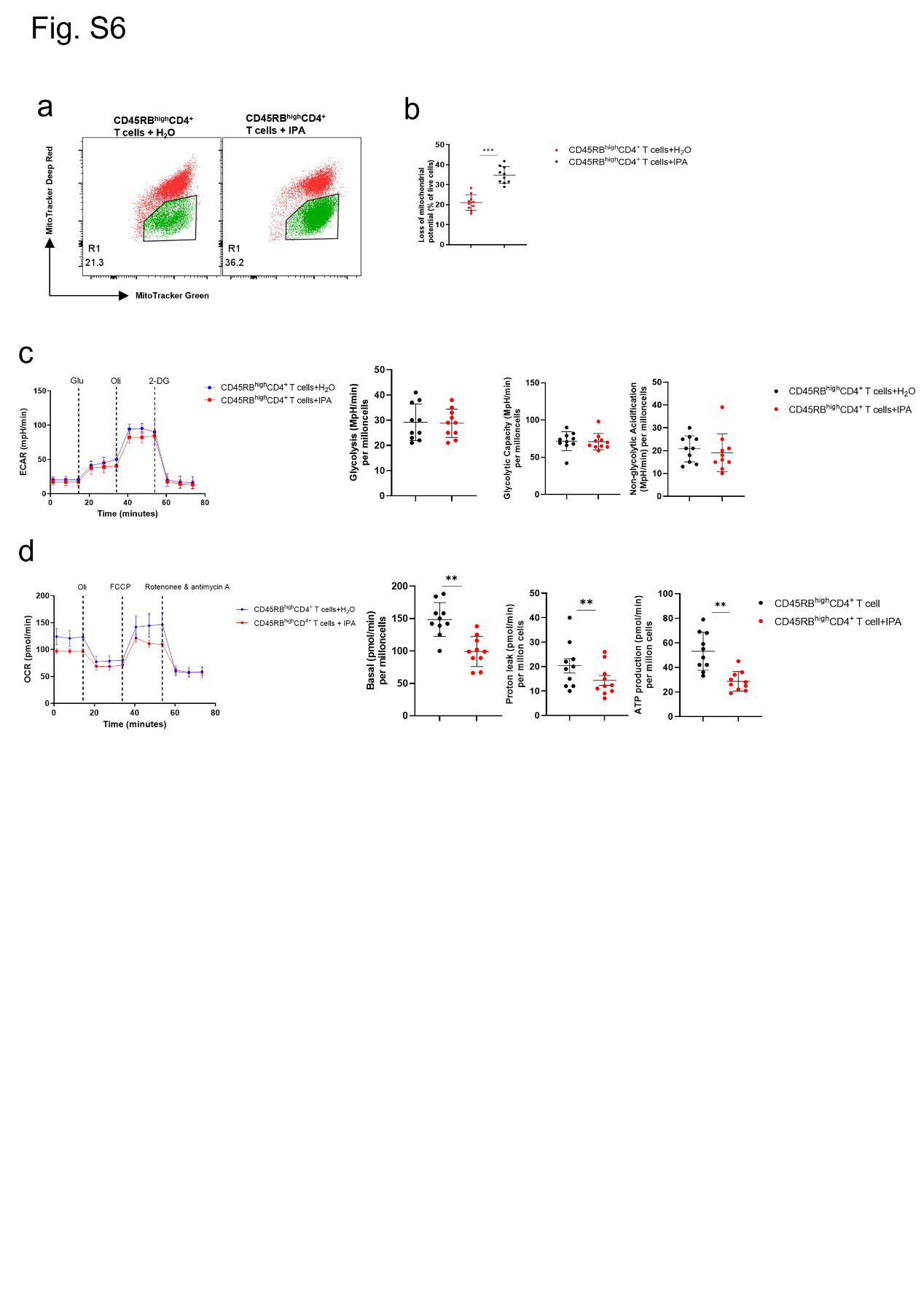


**Figure S6** IPA disrupts mitochondrial integrity and promotes mucosal CD4^+^ T cell apoptosis in chronic murine colitis model. Splenic CD45RB^high^CD4^+^ T cells were isolated from WT mice (n = 5/group) using flow cytometry and injected intraperitoneally into 8-week-old *Rag1*^–/–^ mice (5 × 10^5^ cells/mouse, n = 10/group). (a) LPMCs were harvested from the colons of *Rag1*^–/–^ mice 8 weeks after adoptive transfer of WT CD45RB^high^CD4^+^ T cells, and cells were stained with Mito-Tracker Green and Mito-Tracker Deep Red. The stained cells were then analyzed by flow cytometry, and the results were statistically summarized in (b). (c) The dynamic changes in glycolysis of LP CD4^+^ T cells from colitis mice were measured using a Seahorse extracellular flux analyzer. (d) The dynamic changes in mitochondrial stress of LP CD4^+^ T cells from colitis mice were also assessed using a Seahorse extracellular flux analyzer. The data were represented as the mean ± SEM. Statistical analyses were performed using Student’s *t*-test. * *p* < 0.05, ** *p* < 0.01, *** *p* < 0.001.


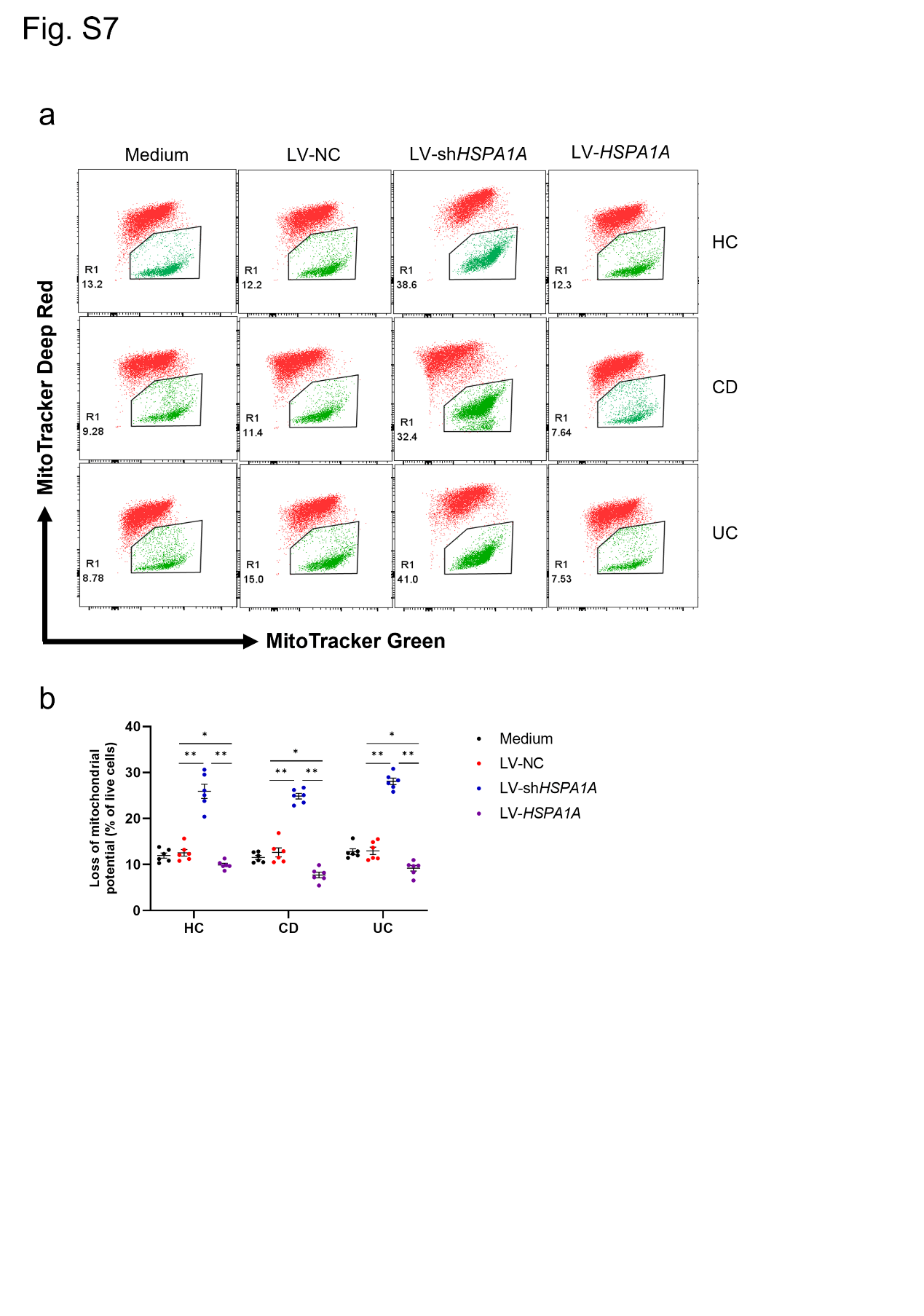


**Figure S7** Overexpression of *HSPA1A* in CD4^+^ T cells suppresses mitochondrial apoptosis. (a) Peripheral blood CD4^+^ T cells (1×10^5^ cells/well) were isolated from 6 healthy individuals, 6 CD patients, and 6 UC patients, respectively, and were then transduced with lentiviruses expressing either shRNA targeting *HSPA1A* (LV-sh*HSPA1A*), *HSPA1A* (LV-*HSPA1A*), or a negative control (LV-NC). These cells were co-cultured with plate-bound anti-human CD3 mAb (5 μg/mL) and soluble anti-human CD28 mAb (2 μg/mL) for 5 days. Following co-culture, the cells were stained with Mito-Tracker Green and Mito-Tracker Deep Red, and then analyzed by flow cytometry. The results were statistically summarized in (b). Statistical analyses were performed using the Student’s *t*-test. * *p* < 0.05, ** *p* < 0.01.


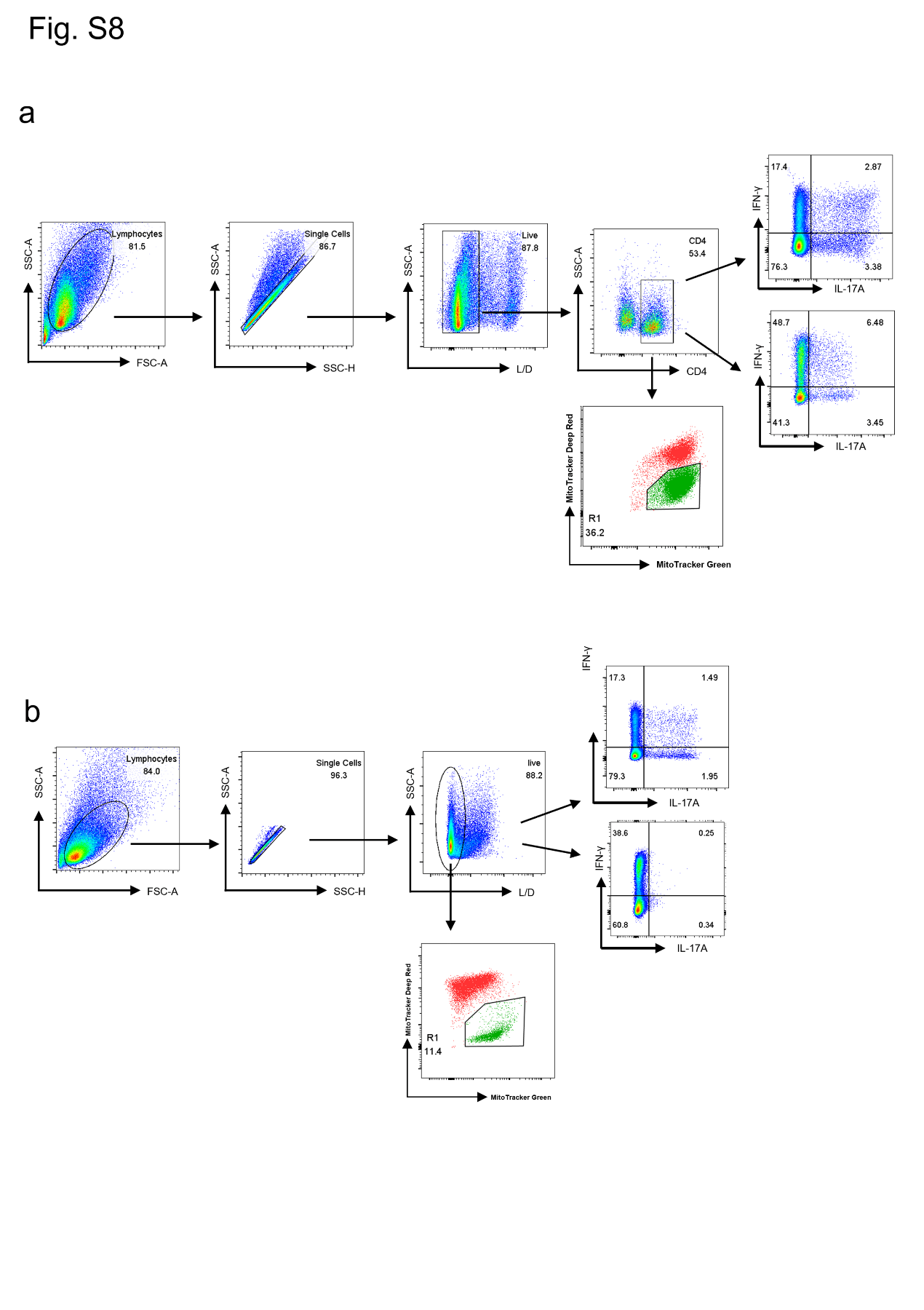


**Figure S8** Gating strategies used in flow cytometry analysis. (a) Gating strategies for mouse CD4^+^ T cells used in Figure 2F, 3F, S1A, and S5A. (b) Gating strategies for human PB-CD4^+^ T cells used in Figure 4A, 4B, 4D, 5F, 6A, 6B, 7A, S4A, S4B, and S6A.

**Supplementary table 1. Clinical characteristics of IBD patients and healthy controls**

| Variable | HC | A-CD | R-CD | A-UC | R-UC |
| --- | --- | --- | --- | --- | --- |
| No. of patients | 59 | 39 | 26 | 35 | 33 |
| Age (years) | 34.92±8.16 | 30.59±7.94 | 31.31±8.54 | 36.83±11.83 | 38.12±10.31 |
| Sex, F (%) | 28 (47.46) | 15 (38.46) | 12 (46.15) | 15 (42.86) | 14 (42.42) |
| Disease duration (months) |  | 31.62±17.95 | 31.65±16.80 | 32.77±20.04 | 34.82±17.98 |
| Smoking |  | 2 | 1 | 2 | 3 |
| Alcohol drinking |  | 3 | 3 | 1 | 1 |
| Current therapy |  |  |  |  |  |
| 5-aminosalicyliclates |  | 3 | 2 | 29 | 26 |
| Immunosuppressants (AZA) |  | 8 | 8 | 9 | 10 |
| Biologics (IFX) |  | 25 | 15 | 18 | 20 |
| Nutritional therapy |  | 2 | 6 | 2 | 3 |
| Disease locations for CD^a^ |  |  |  |  |  |
| L1 (Ileal) |  | 9 | 4 |  |  |
| L2 (Colonic) |  | 2 | 3 |  |  |
| L3 (Ileocolonic) |  | 28 | 19 |  |  |
| L4 (Upper GI tract) |  | 0 | 0 |  |  |
| Disease locations for UC^a^ |  |  |  |  |  |
| E1 (Proctitis) |  |  |  | 2 | 22 |
| E2 (Left-sided colitis) |  |  |  | 6 | 6 |
| E3 (Pancolitis) |  |  |  | 27 | 5 |
| Calprotectin (μg/g) |  | 262.41±58.15 | 91.88±26.26 | 255.74±58.74 | 84.58±29.86 |

CD, Crohn’s disease; HC, Healthy control; A-CD, Active CD; R-CD, CD in remission; F, female; yr, years; mo, months; GI, gastrointestinal; AZA, azathiopurine; IFX, infiliximab

^a^According to the Montreal classification.

**Supplementary table 2. The primer sequences used in qRT-PCR**

|  | **Gene** | | **Forward Primer** | | **Reverse Primer** | |  |
| --- | --- | --- | --- | --- | --- | --- | --- |
| Human | | *GAPDH* | | ACAACTTTGGTATCGTGGAAGG | | GCCATCACGCCACAGTTTC | |
|  |  | *HSPA1A* | | ACCTTCGACGTGTCCATCCTGA | | ATTACACCTCCCAGTGCAGAA | |
|  |  | *IL17A* | | TCCCACGAAATCCAGGATGC | | GGATGTTCAGGTTGACCATCAC | |
|  |  | *RORC* | | GTGGGGACAAGTCGTCTGG | | AGTGCTGGCATCGGTTTCG | |
|  |  | *IFNG* | | TCACCGTCCTAGAAGGATTCAG | | AAACTCTGGTGGTTCAAAAGACA | |
|  |  | *Tbet* | | TTGAGGTGAACGACGGAGAG | | CCAAGGAATTGACAGTTGGGT | |
|  |  | *TNFA* | | GAGGCCAAGCCCTGGTATG | | CGGGCCGATTGATCTCAGC | |
|  |  | *GATA3* | | GCCCCTCATTAAGCCCAAG | | TTGTGGTGGTCTGACAGTTCG | |
|  |  | *IL4* | | CGGCAACTTTGTCCACGGA | | TCTGTTACGGTCAACTCGGTG | |
|  |  | *IL10* | | GACTTTAAGGGTTACCTGGGTTG | | TCACATGCGCCTTGATGTCTG | |
|  |  | *FOXP3* | | GTGGCCCGGATGTGAGAAG | | GGAGCCCTTGTCGGATGATG | |
| Mouse | | *Gapdh* | | AGGTCGGTGTGAACGGATTTG | | GGGGTCGTTGATGGCAACA | |
|  |  | *Hspa1a* | | ACAAGTCGGAGAACGTGCAGGA | | GTTGTCCGAGTAGGTGGTGAAG | |
|  |  | *Il17a* | | TCAGCGTGTCCAAACACTGAG | | CGCCAAGGGAGTTAAAGACTT | |
|  |  | *Rorc* | | CGCGGAGCAGACACACTTA | | CCCTGGACCTCTGTTTTGGC | |
|  |  | *Ifng* | | ATGAACGCTACACACTGCATC | | CCATCCTTTTGCCAGTTCCTC | |
|  |  | *Tbet* | | AGCAAGGACGGCGAATGTT | | GTGGACATATAAGCGGTTCCC | |
|  |  | *Tnfa* | | CAGGCGGTGCCTATGTCTC | | CGATCACCCCGAAGTTCAGTAG | |
|  |  | *Gata3* | | CTCGGCCATTCGTACATGGAA | | GGATACCTCTGCACCGTAGC | |
|  |  | *Il4* | | GGTCTCAACCCCCAGCTAGT | | GCCGATGATCTCTCTCAAGTGAT | |
|  |  | *Il10* | | AGCCTTATCGGAAATGATCCAGT | | GGCCTTGTAGACACCTTGGT | |
|  |  | *Foxp3* | | CACCTATGCCACCCTTATCCG | | CATGCGAGTAAACCAATGGTAGA | |
|  |  | *Tgfb* | | CCACCTGCAAGACCATCGAC | | CTGGCGAGCCTTAGTTTGGAC | |

**Supplementary Table 3. Reagents**

| **Reagent** | **Sources** | **Identifier** |
| --- | --- | --- |
| **Antibodies** |  |  |
| Anti-human FOXP3-PE | BD | 560082 |
| Anti-human IL-10-APC | BD | 562036 |
| Anti-human IL-4-APC | BD | 554486 |
| Anti-human TNF-α-PE-Cy7 | Biolegend | 502929 |
| Anti-human IL-17A-PE | BD | 560438 |
| Anti-human IFN-γ-FITC | Biolegend | 502505 |
| Anti-human CD4-PE-Cy7 | Biolegend | 357410 |
| Anti-human CD4-FITC | Biolegend | 317408 |
| Anti-mouse B220-PE-Cy7 | Biolegend | 103222 |
| Anti-mouse CD3-APC | Biolegend | 100236 |
| Anti-mouse CD8-PE | Biolegend | 100708 |
| Anti-mouse CD4-FITC | Biolegend | 100406 |
| Anti-mouse CD4-PerCP-Cy5.5 | Biolegend | 100434 |
| Anti-mouse IL-4-PE | Biolegend | 504103 |
| Anti-mouse IL-17A-PE-Cy7 | Biolegend | 506922 |
| Anti-mouse IFN-γ-FITC | Biolegend | 505806 |
| Anti-mouse IL-10-PE | Biolegend | 505007 |
| Anti-mouse TNF-α-APC | Biolegend | 506308 |
| Anti-mouse CD45RB-PE | BD | 553101 |
| MitoTracker™ Deep Red FM | Thermofisher | M22426 |
| MitoTracker™ Green FM | Thermofisher | M7514 |
| MitoProbe™ JC-1 | Thermofisher | M34152 |
| Anti-HSP70 | Cell Signaling Technology | 4872S |
| Anti-P-JNK | Santa Cruz Biotechnology | sc-6254 |
| Anti-JNK | Santa Cruz Biotechnology | sc-7345 |
| Anti-P-ASK1 | Abcam | ab278547 |
| Anti-ASK1 | Abcam | ab45178 |
| Anti-CASPASE3 | Cell Signaling Technology | CST9662 |
| Anti-CASPASE9 | Abcam | ab32539 |
| Anti-ACTIN | Santa Cruz | sc-8432 |
| HRP-conjugated Affinipure Goat Anti-Rabbit IgG(H+L) | Proteintech | SA00001-2 |
| HRP-conjugated Affinipure Goat Anti-Mouse IgG(H+L) | Proteintech | SA00001-1 |
| Alexa fluor® 594 conjugated donkey anti-rabbit IgG | Thermofisher | R-37119 |
| **ELISA kit** |  |  |
| HUMAN IL-17A | Biolegend | 433914 |
| HUMAN IFN-γ | Biolegend | 430101 |
| HUMAN TNF-α | Biolegend | 430201 |
| HUMAN-IL-10 | Biolegend | 430601 |
| HUMAN-IL-4 | Biolegend | 430301 |
| **Other Reagents** |  |  |
| Anti-Human CD4 Particles | BD | 557767 |
| Anti-Hu IFN-γ | Thermofisher | 16-7318-85 |
| Anti-Hu CD3 | Thermofisher | 16-0037-85 |
| Anti-Hu CD28 | Thermofisher | 16-0289-85 |
| Anti-Hu IL4 | Thermofisher | 16-7048-85 |
| Recombinant Human IL-12 Protein | R&D | 219-IL-005/CF |
| Recombinant Human TGF-beta 1 Protein | R&D | 240-B-010/CF |
| Recombinant Human IL-6 Protein | R&D | 206-IL-010/CF |
| Recombinant Human IL-1 beta/IL-1F2 Protein | R&D | 201-LB-005/CF |
| Recombinant Human IL-23 Protein | R&D | 1290-IL-010/CF |
| Recombinant Human IL-4 Protein | R&D | 204-IL-010/CF |
