# Supplementary figures and images for: Microbiota-derived IPA alleviates intestinal mucosal inflammation through upregulating Th1/Th17 cell apoptosis in inflammatory bowel disease
Source: Gut Microbes. 2025 Feb 16;17(1):2467235. doi: 10.1080/19490976.2025.2467235 (PMC11834480; doi:10.1080/19490976.2025.2467235)

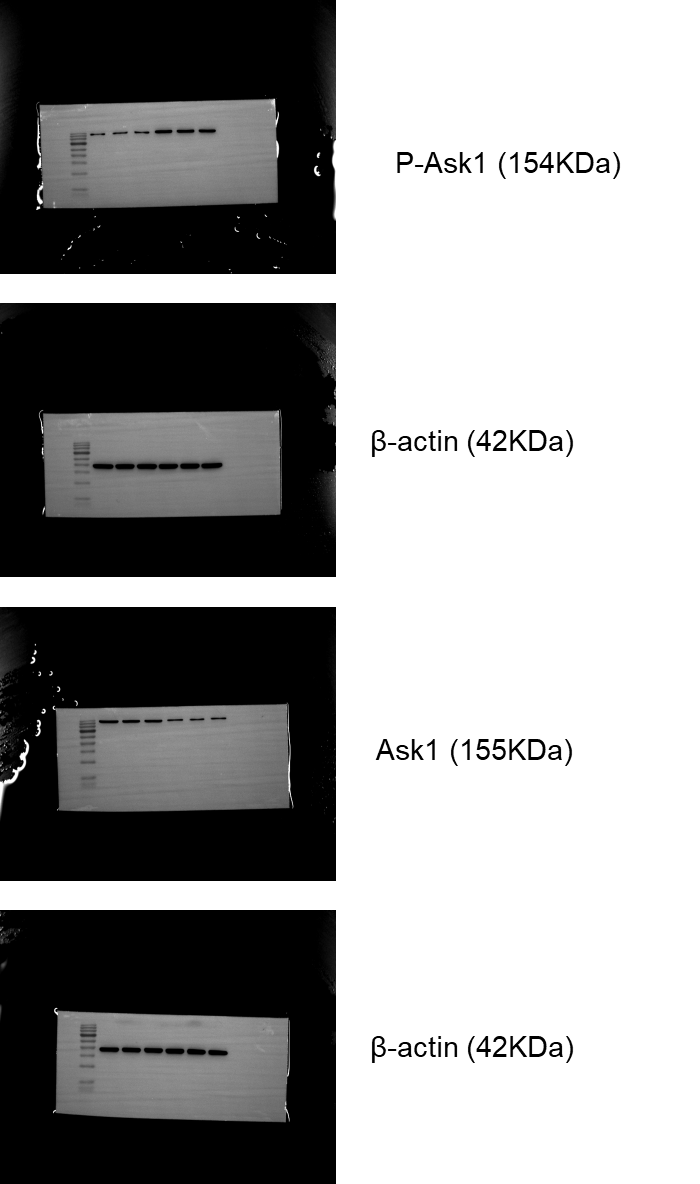


**
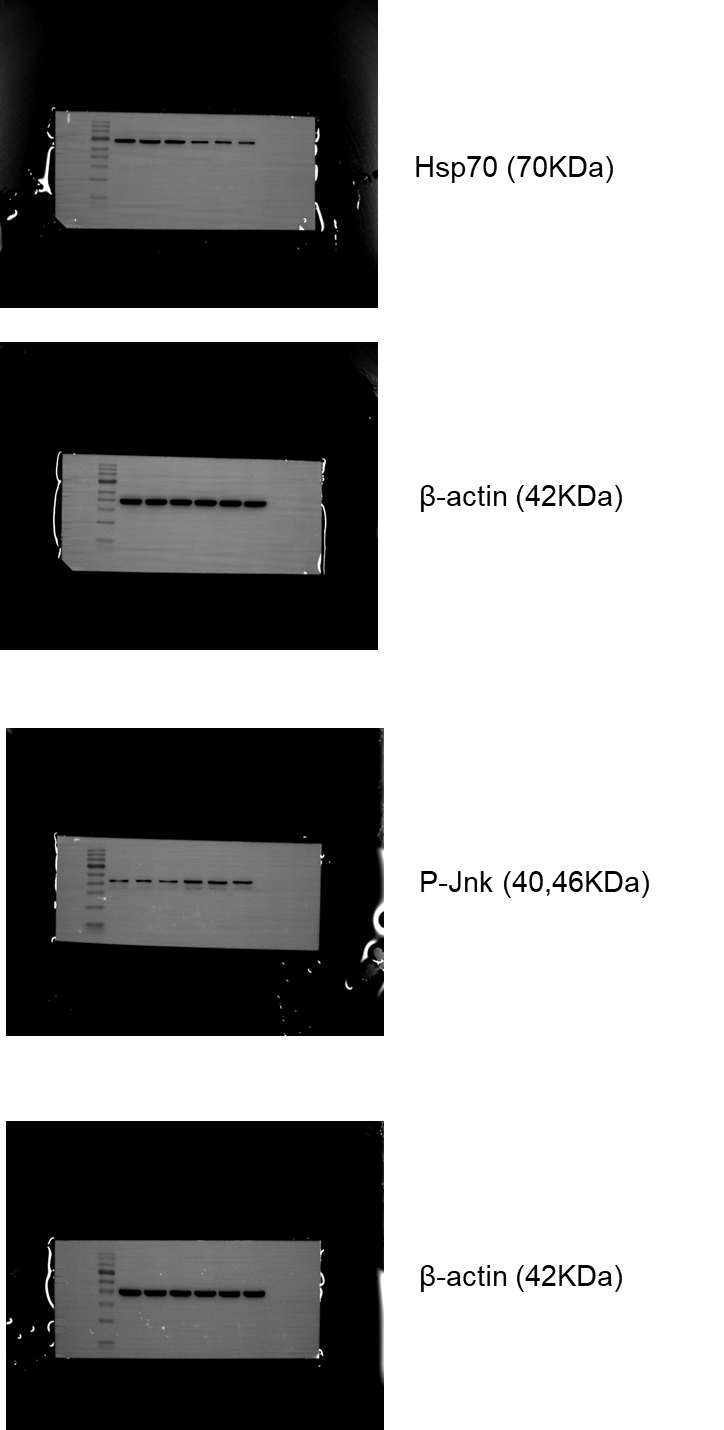
**

**
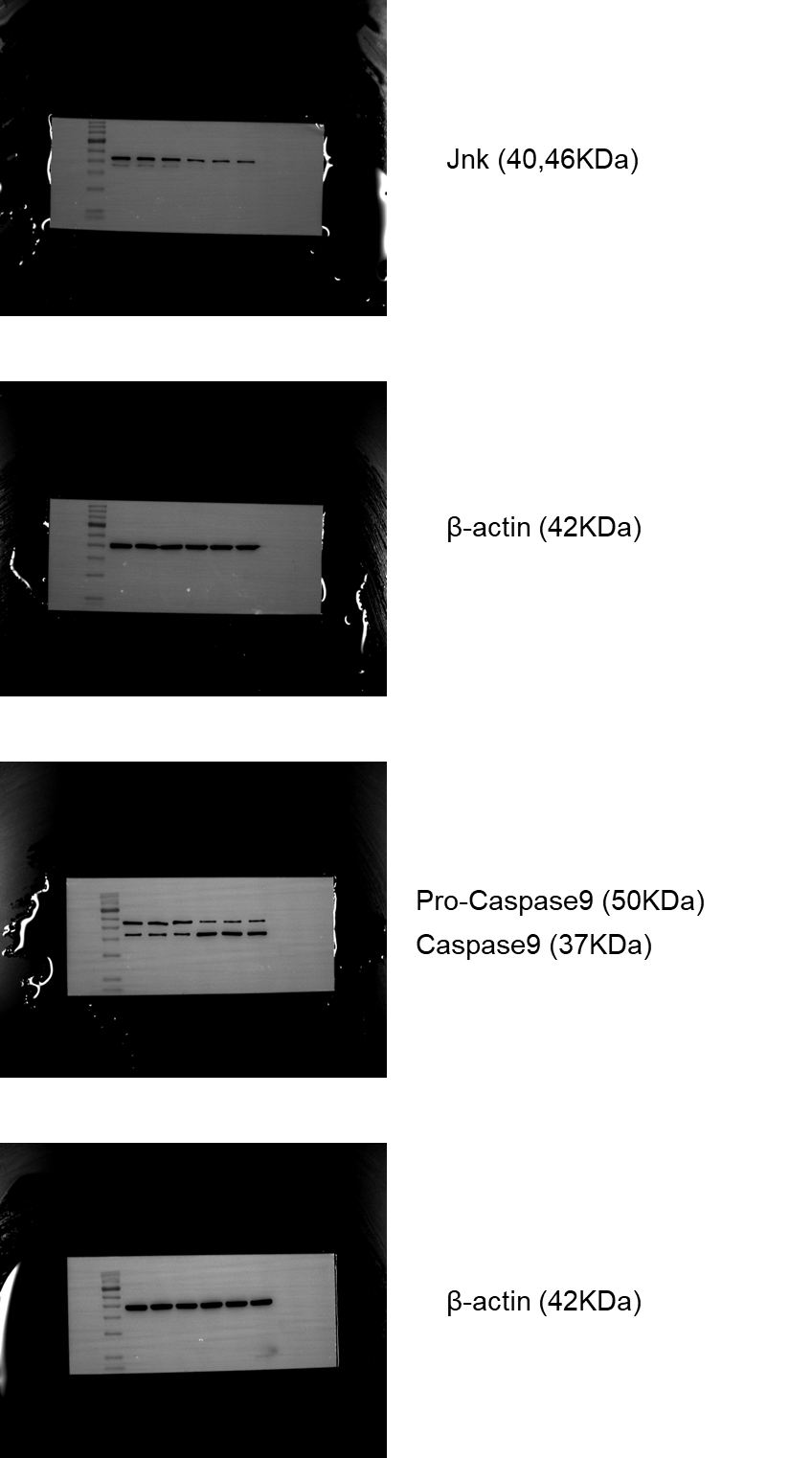
**

**
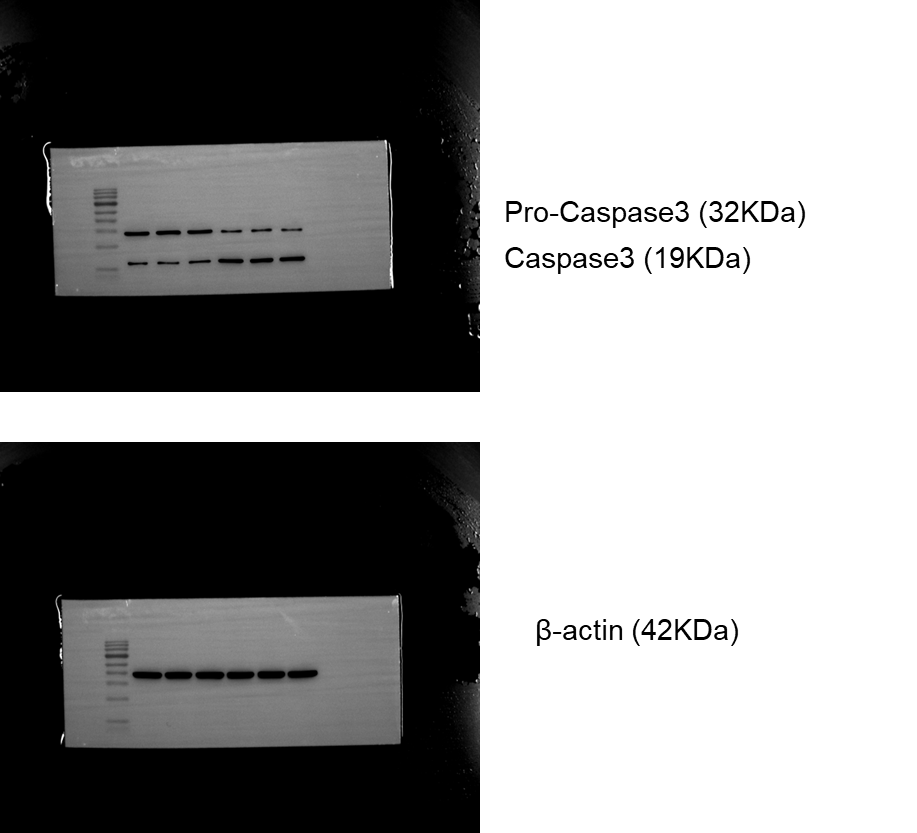
**

Uncropped gels for Figure 6G.

Supplement: Supplemental Material [file KGMI_A_2467235_SM3496.zip › Full length western blots.docx]
